# Nitric oxide‐forming nitrite reductases in the anaerobic ammonium oxidizer *Kuenenia stuttgartiensis*

**DOI:** 10.1002/2211-5463.70086

**Published:** 2025-08-04

**Authors:** Femke J. Vermeir, Lotte W. Nijman, Robert S. Jansen, Laura van Niftrik, Wouter Versantvoort

**Affiliations:** ^1^ Department of Microbiology, Radboud Institute for Biological and Environmental Sciences, Faculty of Science Radboud University Nijmegen The Netherlands; ^2^ Present address: Chair of Bioinorganic Chemistry Heinrich‐Heine‐Universität Düsseldorf Düsseldorf Germany

**Keywords:** anaerobic ammonium oxidation, anammox bacteria, hydroxylamine oxidoreductase (HAO)‐like protein HAOr, *Kuenenia stuttgartiensis*, NirS, nitrite reductase

## Abstract

Anaerobic ammonium‐oxidizing (anammox) bacteria contribute to the global nitrogen cycle by removing fixed nitrogen from the environment. They do so via the anaerobic oxidation of ammonium to dinitrogen gas, with nitrite as terminal electron acceptor. The first step in this so‐called anammox reaction is the proposed conversion of nitrite to nitric oxide by a nitrite reductase. There is an unusual diversity and redundancy in anammox nitrite reductases and to gain more insight into the puzzling redundancy and diversity, we investigated the putative reductases in the model anammox species ‘*Candidatus* Kuenenia stuttgartiensis’. The genome of this model anammox species encodes for three putative nitrite reductases and we investigated which of these is or are active in *K. stuttgartiensis* strain MBR1. Active nitric oxide‐producing nitrite reductases were enriched from *K. stuttgartiensis* cells via fast protein liquid chromatography. Nitric oxide production by the enriched nitrite reductases was followed with membrane inlet mass spectrometry. Combining the activity assays with proteomics analysis indicated that the soluble nitrite reductases NirS and HAOr most strongly correlated with enzyme activity. This indicates that *K. stuttgartiensis* strain MBR1 employs two distinct nitrite reductases to keep its nitric oxide pool replenished. Containing two different nitrite reductases could improve the adaptability of *K. stuttgartiensis* to changes in environmental nitrite concentrations.

## Graphical abstract

Anammox bacteria remove fixed nitrogen from their environment via anaerobic ammonium oxidation (anammox) with nitrite as electron acceptor and dinitrogen gas as product. The first anammox step is the conversion of nitrite to nitric oxide by nitrite reductase. Here we found that anammox bacterium *Kuenenia stuttgartiensis* uses two nitrite reductases, NirS and HAOr, to keep its nitric oxide pool replenished.

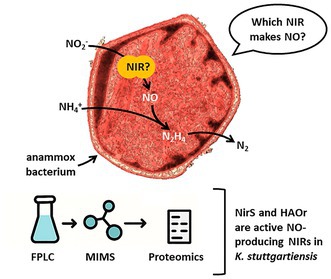

**Abbreviations**
ABC: ammonium bicarbonate
Anammox: anaerobic ammonium oxidation
BCA: bicinchoninic acid
BSA: bovine serum albumin
CE: cell‐free extract
Census MBR: census match between runs
dda‐PASEF: default data‐dependent acquisition–parallel accumulation serial fragmentation
FPLC: fast protein liquid chromatography
FT: flow through
HAO: hydroxylamine oxidoreductase
HOX: hydroxylamine dehydrogenase
LFQ: label‐free quantitation
MBR: membrane bioreactor
MF: membrane protein fraction
MIMS: membrane inlet mass spectrometry
NirS: nitrite reductase S
NXR: nitrite oxidoreductase
PaSER: parallel search engine in real‐time
r: Pearson correlation score
R/b: Rieske/cytochrome *b* complex
RPKM: reads per kilobase per million mapped reads
SDS: sodium dodecyl sulfate
SDS/PAGE: sodium dodecyl sulfate polyacrylamide gel electrophoresis
SF: soluble protein fraction
SPD: samples per day
tims‐TOF MS: trapped ion mobility spectrometry–quadrupole time‐of‐flight mass spectrometer

Anaerobic ammonium‐oxidizing (anammox) bacteria fulfill an important role in the global nitrogen cycle by releasing fixed nitrogen from oxygen‐limited environments such as agricultural soils and oceans [1, 2]. In addition to their significance in natural environments, anammox bacteria are applied in wastewater treatment plants for the cost‐effective removal of nitrogen [3, 4].

Anammox bacteria have a chemolithoautotrophic lifestyle and perform the catabolic anammox reaction in a dedicated intracellular compartment called the anammoxosome [5, 6]. The anammox reaction proceeds via three coupled reactions interconnected by a cyclic electron flow and two reactive intermediates: nitric oxide and hydrazine [7]. First, nitrite reductase catalyzes the one‐electron reduction of the substrate nitrite to nitric oxide [8, 9]. Then, hydrazine synthase produces hydrazine via the combination of nitric oxide and the substrate ammonium, with the input of three electrons [10, 11]. Finally, hydrazine dehydrogenase oxidizes hydrazine to the product dinitrogen gas, releasing four electrons [12]. Although the genes/enzymes that function as hydrazine synthase and hydrazine dehydrogenase in anammox bacteria are known, the identity of nitrite reductase remains ambiguous.

Anammox bacteria are cultivated in bioreactor systems as enrichment cultures that can reach up to 95% purity [13]. Enrichments and molecular approaches have identified various anammox genera, including ‘*Candidatus* Brocadia’ [14], ‘*Candidatus* Kuenenia’ [15], ‘*Candidatus* Anammoxoglobus’ [16], ‘*Candidatus* Jettenia’ [17], ‘*Candidatus* Anammoxibacter’ [18], and ‘*Candidatus* Scalindua’ [19]. New families and genera continue to be discovered [20, 21, 22]. In enrichment cultures, anammox bacteria grow slowly with doubling times between 1.8 and 12 days, depending on the bioreactor system used [23, 24, 25, 26]. Due to slow growth, the absence of pure cultures, and a lack of standard cultivation techniques, there is currently no genetic modification toolbox available to facilitate gene and protein function studies in anammox bacteria. Therefore, our current understanding of anammox physiology and biochemistry is largely obtained from enzyme assays, transcriptomics, proteomics, and electron microscopy studies using *Kuenenia stuttgartiensis* as the anammox model species.

Different anammox genera encode different putative nitrite reductases in their genome. The genomes of *K. stuttgartiensis* and *Scalindua profunda* contain the nitrite reductase S (*nirS*) gene encoding a cytochrome *cd*_1_ type nitrite reductase. However, expression of this gene (kuste4136, GenBank CAJ74898) in *K. stuttgartiensis* is relatively low (1.000 RPKM (reads per kilobase per million mapped reads) [27]) compared to the expression of the genes encoding the other anammox reaction enzymes, that is, hydrazine synthase (kuste2859, kuste2860 and kuste2861, GenBank CAJ73611, CAJ73612 and CAJ73613) and hydrazine dehydrogenase (kustc0694 and kustd1340, GenBank CAJ71439 and CAJ72085) (12.000–28.000 RPKM and 25.000 RPKM, respectively) [10, 27]. In contrast, *nirS* is prominently expressed in *S. profunda* [28]. The genomes of *Jettenia caeni* and *Jettenia asiatica* do not contain the *nirS* gene but encode for the copper‐containing nitrite reductase NirK instead [29, 30, 31]. *Brocadia* species seemingly lack genes encoding for canonical nitrite reductase [32, 33, 34], although a *nirK* gene was reported in the genome of *B. carolinensis* [35]. Due to these genomic variations in canonical nitrite reductase genes, it was postulated that anammox bacteria might harbor a novel and perhaps anammox‐specific nitrite‐reducing enzyme preserved throughout the different genera.

A candidate for this putative anammox nitrite reductase is the hydroxylamine oxidoreductase (HAO)‐like protein HAOr. HAOr is encoded by kustc0458 (GenBank CAJ71203) and redox partner kustc0457 (GenBank CAJ71202) in *K. stuttgartiensis* and is one of the HAO‐like proteins that is present in all anammox genera ([36] and for a specific overview of HAO‐like proteins see fig. 2A in [7]). HAOr was isolated from *K. stuttgartiensis* and reported to reduce nitrite to nitric oxide with a rate of 0.52 nmol·min^−1^·mg^−1^ protein [9]. This is relatively slow compared to the potential rate of nitrite reductases NirS and NirK in denitrifiers that produce up to 4.15 and 380 μmol·min^−1^·mg^−1^ protein, respectively [37]. However, in *K. stuttgartiensis*, HAOr was detected in substantial amounts (4700 RPKM [27, 38]) which could potentially meet the nitric oxide demands of hydrazine synthase. Despite its low activity, HAOr is a plausible candidate for the nitrite reductase shared among all anammox genera. Interestingly, a close homolog of HAOr, Kuste4574 (GenBank CAJ75336), has been identified. Kuste4574 is a component of one of the three Rieske/cytochrome *b* complexes (kuste4569–4574 (R/*b*‐3)) embedded in the anammoxosome membrane [36, 39] and exhibits a high expression level (1800 RPKM). Similar to HAOr, this enzyme may produce nitric oxide from nitrite. However, experimental evidence confirming Kuste4574 activity is currently lacking [27, 36].

In expression studies [27], *K. stuttgartiensis* HAOr and NirS both respond to changing nitrogen‐species concentrations in the environment. When *K. stuttgartiensis* was grown in nitrite‐limited conditions, the *nirS* and *haor* genes were significantly upregulated by 10‐ and 2.5‐fold, respectively. Conversely, another study showed that the *nirS* and *haor* genes were strongly downregulated in an environment with ammonium and nitric oxide as substrates (instead of ammonium and nitrite), by 25‐ and 28‐fold, respectively [40]. These data suggest that both nitrite reductases are of functional and physiological relevance in *K. stuttgartiensis* cells.

Based on current genomic and biochemical knowledge, anammox bacteria have various potential nitrite reductases at their disposal to produce nitric oxide for the activation of ammonium into hydrazine. For the model anammox species *K. stuttgartiensis*, these are NirS, HAOr (both soluble) and potentially Kuste4574 (membrane‐bound). In order to identify the physiological nitrite reductase in *K. stuttgartiensis*, we enriched active nitric oxide‐producing nitrite reductases from *K. stuttgartiensis* strain MBR1 cells via fast protein liquid chromatography. Nitric oxide production by the enriched nitrite reductases was followed with membrane inlet mass spectrometry, and nitrite reductases were identified using proteomics. Combining the activity assays with proteomics analysis indicated that the soluble nitrite reductases NirS and HAOr most strongly correlated with enzyme activity.

## Materials and methods

### Cultivation of *Kuenenia stuttgartiensis*

*‘Candidatus* Kuenenia stuttgartiensis’ strain MBR1 [41] was grown as planktonic cells in a 12‐L membrane bioreactor (MBR) at 33 °C with an OD_600_ of ~ 1.2 (Applikon B.V., Schiedam, the Netherlands), as described by Kartal *et al*. [42]. In brief, the bioreactor was operated at 33 °C and kept anoxic by continuous flushing of the bioreactor and medium vessel with argon/carbon dioxide (95/5%, 10 mL·min^−1^). The carbon dioxide in the supplied gas was sufficient to maintain the pH in the bioreactor between 7.0 and 7.4. Planktonic biomass was removed via a direct connection to a neighboring vessel at 1.1 L·day^−1^, resulting in a doubling time in the bioreactor culture of 10 days. The collected biomass was kept at room temperature under continuous sparging with dinitrogen gas to keep it anaerobic for preparation of cell‐free extract.

### Preparation of cell‐free extract, soluble, and membrane protein fractions

For preparation of cell‐free extract and soluble and membrane protein fractions, all steps were carried out at 4 °C. To collect cells, 1200 mL anaerobically collected *K. stuttgartiensis* biomass of the bioreactor described above was centrifuged at 10 000 ***g*** for 10 min (Sorvall centrifuge, fixed angle). The cell pellet was resuspended in 13 mL 20 mm MOPS, 150 mm NaCl, pH 7.5 and transferred to a French press cell. Bacterial cells were broken by passing them once through the French press at 138 MPa (American Instrument Company, Hartland, WI, USA). Whole cells and cell debris were removed by centrifugation at 5000 ***g*** for 20 min (Allegra X‐15R, swinging bucket rotor; Beckman Coulter, Indianapolis, IN, USA) thus obtaining the cell‐free extract.

Of the cell‐free extract, 1 mL was set apart for nitrite reductase activity measurements described below. To separate membranes and soluble proteins, 6 mL of the cell‐free extract was subjected to ultracentrifugation at 153 300 ***g*** for 1 h (Optima‐XE‐90, Fixed‐Angle 90 Ti rotor; Beckman Coulter). After centrifugation, the supernatant contained the soluble protein fraction and the pellet contained membranes and membrane proteins. The pellet was homogenized in ~ 6 mL 20 mm MOPS, 150 mm NaCl, pH 7.5 with a glass potter device, thus obtaining the membrane protein fraction.

All samples were concentrated using 10 kDa molecular weight cutoff spin filters centrifuged at 4000 ***g*** (Sartorius, Göttingen, Germany) and their protein concentrations were measured with the Bicinchoninic acid (BCA) assay (Pierce BCA Protein Assay Kit; Thermo Scientific, Waltham, MA, USA). Sodium dodecyl sulfate (SDS; Sigma‐Aldrich, St. Louis, MI, USA, 2% final concentration) was added to all samples and to the calibration curve samples (with bovine serum albumin, BSA, as standard), prior to protein quantification. SDS‐dissolved membranes present in the cell‐free extract and membrane protein fraction, preventing their interference in the BCA assay [43].

As a control, it was evaluated whether membranes still present in the cell‐free extract and membrane protein fraction influenced nitrite reductase activity measurements. To this end, membranes were dissolved by incubating 1 mL of cell‐free extract, membrane protein fraction, and soluble protein fraction with *n*‐dodecyl β‐d‐maltoside (Thermo Scientific; 1% final concentration) in a rotating mixer overnight. The following day, insolubilized membranes, and insolubilized membrane proteins were pelleted via ultracentrifugation at 142 600 ***g*** for 50 min (Optima‐XE‐90, Fixed‐Angle 90 Ti Rotor; Beckman Coulter). The remaining supernatant of cell‐free extract contained soluble proteins, solubilized membrane proteins, and membrane‐associated proteins. The supernatant of the soluble protein fraction contained only soluble proteins. The supernatant of the membrane protein fraction contained solubilized membrane proteins and membrane‐associated proteins. Samples were concentrated using 10 kDa molecular weight cutoff spin filters centrifuged at 4000 ***g*** (Sartorius), and protein concentrations were measured with the BCA assay.

### Enrichment of soluble nitrite reductases

Note to reader: it is highly recommended to read the results section guided by Fig. 8 to keep a clear overview of the experiments and fractions.

#### Initial separation of soluble proteins via low‐resolution anion‐exchange column chromatography with Q‐Sepharose

Soluble protein fraction was prepared as described above. Soluble proteins (10 mL) were separated with fast protein liquid chromatography (FPLC) on an Äkta Purifier (GE Healthcare, Chicago, IL, USA) with a 70 mL column packed with Q‐Sepharose (XK 26/20; GE Healthcare/Cytiva). The Q‐Sepharose column was equilibrated with 1.5 column volumes of 20 mm Tris/HCl buffer, pH 8.0 at a flow rate of 5 mL·min^−1^. Nonbinding proteins were collected in the flow‐through fraction. When the UV signal and conductivity were stable, proteins were eluted in seven‐column volumes via a linear gradient from 0 to 1 m NaCl and collected in 10 mL fractions. Elution of proteins was monitored at 280 nm. Collected fractions were buffer‐exchanged to 20 mm MOPS, 150 mm NaCl buffer, pH 7.5, and concentrated using 10 kDa molecular weight cutoff spin filters centrifuged at 4000 ***g*** (Vivaspin 20; Sartorius Stedim Lab Ltd) for nitrite reductase activity measurements. Selected fractions that eluted from the Q‐Sepharose column were pooled and further separated based on specific activity and their contribution to the total nitric oxide production.

#### High‐resolution anion‐exchange column chromatography with source Q15

Pooled protein fractions 9–17 (sample B) and 11–14 (sample B1) from the low‐resolution anion‐exchange column were further separated on a 1.7 mL Source 15Q column (4.6/100 PE; Cytiva) equilibrated with 20 column volumes of 20 mm Tris/HCl buffer, pH 8.0, with a flow rate of 2 mL·min^−1^. After the UV signal and conductivity were stable, proteins were eluted in 40 column volumes via a linear gradient from 0 to 1 m NaCl. Collected fractions of 1 mL were either pooled based on the UV signal and buffer‐exchanged to 20 mm MOPS, 150 mm NaCl buffer, pH 7.5 using 10 kDa spin filters or directly buffer‐exchanged. Protein fractions were concentrated using 10 kDa spin filters centrifuged at 4000 ***g*** to measure nitrite reductase activity.

#### Mixed‐mode column chromatography with hydroxyapatite

Collected flow‐through of the low‐resolution anion‐exchange column was concentrated and buffer‐exchanged to 20 mm potassium phosphate buffer, pH 7.0 using 10 kDa spin filters. Concentrated flow‐through was loaded onto a 5 mL ceramic hydroxyapatite column (EconoFit CHT Type II, 40 μm Column; Bio‐Rad, Hercules, CA, USA) equilibrated with five column volumes of 20 mm potassium phosphate buffer, pH 7.0 with a flow rate of 1.5 mL·min^−1^. Proteins were eluted in nine column volumes via a linear gradient of 20–500 mm potassium phosphate and collected in fractions based on UV signal. The fractions were buffer‐exchanged to 20 mm MOPS, 150 mm NaCl buffer, pH 7.5 and concentrated using 10 kDa molecular weight cutoff spin filters centrifuged at 4000 ***g*** to measure nitrite reductase activity.

### Determination of protein concentration and sample purity

Protein concentrations were determined in the fractions with the absorbance of each protein sample at wavelengths 260 and 280 nm on a Cary 60 spectrophotometer (Agilent Technologies, Santa Clara, CA, USA) with 20 mm MOPS, 150 mm NaCl buffer, pH 7.5 as baseline. From these values, protein concentrations were calculated with the formula: concentration (mg·mL^−1^) = *A*_280_ × 1.55 − *A*_260_ × 0.76 [44].

Purity of the protein samples was assessed with sodium dodecyl sulfate polyacrylamide gel electrophoresis (SDS/PAGE) [45] with a 10% resolving and 4% stacking gel. The gel was imaged on a ChemiDoc MP Imaging System (Bio‐Rad).

### Membrane‐inlet mass spectrometry activity assays

Nitrite reductase activity in the protein fractions was measured via ^15^N‐labeled nitric oxide production followed with membrane inlet mass spectrometry (MIMS; HPR40, Positive Ion Counting detector; Hiden Analytical, Warrington, UK). The electron emission current was set to 400 μA and the measurement frequency to 1 s^−1^. Assays were performed in a custom‐built 5.16 mL MIMS chamber [46] filled with 20 mm MOPS, 150 mm NaCl buffer, pH 7.5. The MIMS chamber was made anaerobic prior to measurements by flushing it with Argon, and the temperature was maintained at 30 °C throughout the measurements. All components of the assay were made anaerobic before they were added with a gastight syringe (Hamilton, Reno, Nevada, USA) to the MIMS chamber. Assays consisted of 200 μm phenazine ethosulfate (MP Biomedicals, Santa Ana, CA, USA , LLC) and 200 μm l‐ascorbic acid (Sigma) for the donation and shuttling of electrons, and 6–10 μg protein. The reaction was started by the addition of 77 μm ^15^N‐labeled nitrite (99%; Cambridge Isotope Laboratories, Tewksbury, MA, USA). For calibration curves, a nitric oxide stock solution was made by flushing anaerobic ultrapure water (PURELAB Chorus 1 Veolia, Paris, France) with 4% nitric oxide for 15 min. The nitric oxide stock concentration was calculated with the formula: concentration (m) = *H* ^cp^ × *p*, in which *H* ^cp^ for nitric oxide is 0.0019 m·atmosphere^−1^ [47] and *p* is the partial pressure in the stock solution of nitric oxide. For each calibration level, nitric oxide was added to the MIMS chamber. Rates of nitric oxide production were determined by a linear regression fit to the initial linear part of the graph with originpro 2020b (OriginLab, Northampton, MA, USA).

### Proteomics

To identify nitrite reductases present in active protein fractions, proteomics analysis was performed. Incubations were performed at room temperature unless stated otherwise. For proteins collected from the low‐resolution anion‐exchange column and the high‐resolution anion‐exchange column, samples were prepared as follows: 8 m urea was added to the sample (1 : 4 (v/v)), then 10 mm dithiothreitol was added (1 : 500 (v/v)) and the sample was incubated for 20 min at room temperature. Subsequently, 50 mm 2‐chloroacetamide was added (1 : 500 (v/v)) and the sample was incubated for 20 min in the dark at room temperature. Finally, proteins were digested overnight at 37 °C with trypsin (1 : 50 (w/w)). Samples were stored at −20 °C until analysis.

Protein fractions collected from the mixed‐mode column were diluted 1 : 1 (v/v) with 8 m Urea in 10 mm Tris, pH 8.0. Samples were incubated for 30 min with 1 μL 10 mm dithiothreitol per 50 μg protein in the sample. The sample volume was doubled by adding 50 mm 2‐chloroacetamide in 50 mm ammonium bicarbonate (ABC) (1 : 1 (v/v)) and samples were incubated for 20 min in the dark. Subsequently, samples were digested overnight at 37 °C with trypsin (1/50 (w/w)) and diluted 1 : 1 (v/v) with 2% TFA the following day. Protein fractions of 2.5 mL were desalted with OMIX C18 100 μL tips (Agilent Technologies) that were prepared by washing them two times with 100 μL 0.1% formic acid in acetonitrile, followed by two times washing with 100 μL 0.1% formic acid in ultrapure water. Protein samples were aspirated and expelled five times in order to allow proteins to bind to the OMIX C18 tip. Tips were again washed two times with 100 μL 0.1% formic acid in ultrapure water, after which proteins were eluted with 100 μL 0.1% formic acid in acetonitrile. The eluted protein samples were dried and concentrated in a vacuum centrifuge (Savant ISS110; Thermo Scientific) and resuspended in 20 μL 0.1% formic acid in ultrapure water. Samples were stored at −20 °C until analysis.

Analysis of the protein fractions was performed at the Radboudumc Proteomics Center. Protein samples were analyzed by nanoflow liquid chromatography (Evosep One; Evosep Biosystems, Odense, Denmark) coupled to a trapped ion mobility spectrometry–quadrupole time‐of‐flight mass spectrometer (tims‐TOF MS Pro2; Bruker Daltonics) via a nanoflow electrospray ionization source (CaptiveSprayer; Bruker Daltonics, Billerica, MA, USA). Tryptic peptides (for all low‐ and high‐resolution anion‐exchange column fractions: 200 ng and for the mixed‐mode column fractions 1 and 4: 200 ng, fraction 2: 100 ng and fraction 3: 10 ng) were separated by C18 reversed phase liquid chromatography (Evosep EV1106 30SPD endurance column; 150 mm length × 0.150 mm internal diameter, 1.9 μm C18AQ particles) using the preprogrammed 30 samples per day (30SPD) Evosep One method. The mass spectrometer was operated in positive ionization mode using the default data‐dependent acquisition–Parallel Accumulation Serial Fragmentation (dda‐PASEF) [48] instrument method: 0.6–1.6 1/K0 mobility range, 100–1700 *m/z* mass range, 100 ms accumulation time, 100 ms ramp time, 10 PASEF ramps per duty cycle, 20 K target PASEF intensity, 20–59 eV linear scaled collision energy between 0.6 1/K0 and 1.6 1/K0, dynamic exclusion enabled for 0.4 min.

Acquired spectra from samples of the low‐ and high‐resolution anion‐exchange column were streamed directly to ProteoScape (v2025b; Bruker Daltonics) for protein identification and label‐free quantitation against the *K. stuttgartiensis* protein sequence database (UniProt entry KSMBR1) using the following settings: Spectronaut v19 directDIA+ (Fast) workflow, 0.2 precursor PEP cutoff, 0.01 precursor *Q*‐value cutoff, 0.01 protein *Q*‐value cutoff global, 0.01 protein *Q*‐value cutoff, 0.75 protein PEP cutoff, full tryptic specificity, allowed up to 2 missed cleavages, carbamidomethyl (C) as fixed modification and oxidation (M) as variable modifications, protein group specific peptides were used for quantitation.

Acquired spectra from samples of the mixed‐mode column were streamed directly to the Parallel Search Engine in Real‐time (PaSER v2023; Bruker Daltonics) box for real‐time database searching of acquired MS/MS spectra against the provided custom *K. stuttgartiensis* protein sequence database using the following settings: ProLuCID database search algorithm [49], 20 p.p.m. precursor ion mass tolerance, 30 p.p.m. fragment ion mass tolerance, full tryptic enzyme specificity, allowed up to two missed cleavages, carbamidomethyl (C) as a fixed modification, deamidation (NQ) and oxidation (M) as variable modifications, TIMScore enabled, and 1% protein‐level false discovery rate validation using DTA Select. Label‐free quantitation (LFQ) was performed in PaSER using Census Match Between Runs (Census MBR) [50] with the following parameters: 15 p.p.m. mass accuracy, 0.03 1/K0 mobility tolerance, 30 s retention time tolerance, 3 isotope traces, 0.7 correlation threshold, and 0.2 root mean square averaging error.

### Calculations

For label‐free quantification of proteins, the relative abundance of each protein was estimated by summing the intensity values of all detected peptide ions corresponding to that protein. Relative abundance was normalized on the injected amounts of peptides. Pearson correlations between specific enzyme activities (obtained by MIMS) and relative abundance were calculated in Excel.

Specific activity of the samples was calculated with the production of ^15^N‐labeled nitric oxide in nmol·min^−1^ divided by the amount of protein in the sample. From this, the purity fold per sample was calculated by dividing the specific activity by the specific activity measured in cell‐free extract or soluble protein fraction. The yield of nitrite reductase activity per fraction was determined by dividing the total activity in the fraction by the total activity of cell‐free extract or soluble protein fraction.

### Figures

Figures were made in rstudio version 4.4.1 (Boston, MA, USA) with the ggplot2, readxl, ggrepel, and cowplot packages and in Adobe Illustrator 2024 (San Jose, CA, USA).

## Results

### Soluble nitrite reductase produces most nitric oxide in *K. stuttgartiensis* strain MBR1

To determine whether active nitrite reductase is soluble or membrane‐bound, we measured nitrite reductase activity in crude cell‐free extract, membrane protein fraction, and soluble protein fraction. Nitrite reductase activity was followed continuously via ^15^N‐labeled nitric oxide production from ^15^N‐labeled nitrite using MIMS. Because the physiological electron donor for anammox nitrite reductases is unknown, we chose ascorbate and phenazine ethosulfate as electron donor and carrier, respectively. These electron carriers have the ability to donate electrons to various proteins in *in vitro* enzyme assays. Moreover, their moderate midpoint potentials ($E_{0}^{\prime }$ = +80 mV for ascorbate and +55 mV for phenazine ethosulfate) make them suitable for direct nitric oxide measurement via MIMS [9].

**Fig. 1:**
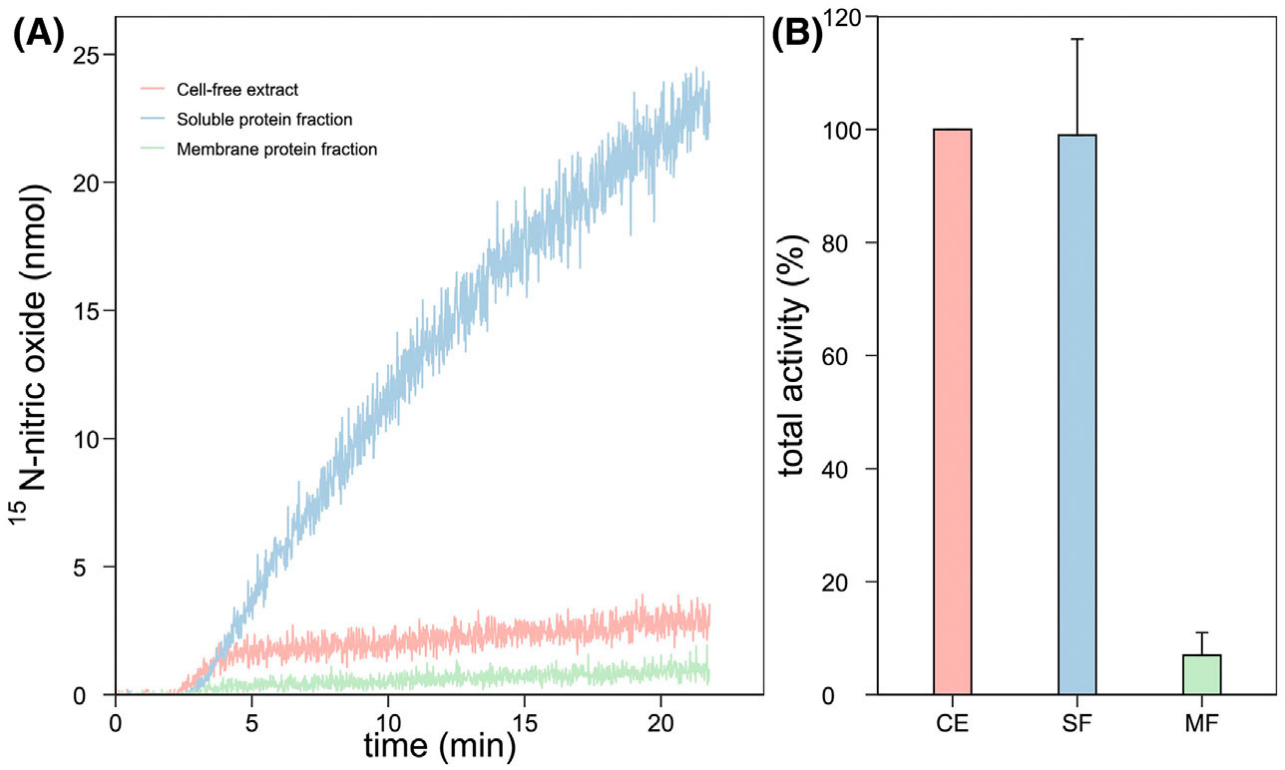
Nitrite reduction to nitric oxide in *Kuenenia stuttgartiensis* cell‐free extract and protein fractions. (A) ^15^N‐labeled nitric oxide production from ^15^N‐labeled nitrite in protein fractions was followed with membrane inlet mass spectrometry. (B) Soluble proteins account for the majority of nitric oxide production from nitrite in *K. stuttgartiensis*. Activity assays contained 200 μm ascorbate and phenazine ethosulfate, and 6–10 μg protein in 20 mm MOPS, 150 mm NaCl buffer, pH 7.5. The reaction was started with 77 μm ^15^N‐nitrite and carried out at 30 °C. CE, cell‐free extract; MF, membrane protein fraction; SF, soluble protein fraction. Data are presented as mean ± SD. For A, *n* = 1 biological replicate, and for B, *n* = 3 biological replicates.

Of the total nitrite reductase activity measured in *K. stuttgartiensis* cell‐free extract, the soluble protein fraction accounted for 99 ± 17% of the activity, whereas 7 ± 4% of the activity could be attributed to the membrane protein fraction (Fig. 1A,B, see also Table S1 and Fig. 8). Soluble proteins produced nitric oxide at 12 ± 2 nmol·min^−1^·mg^−1^ protein, and nitrite reductase was two times enriched compared to cell‐free extract. Membrane proteins produced nitric oxide at a rate of 0.8 ± 0.5 nmol·min^−1^·mg^−1^ protein, and nitrite reductase was not enriched compared to cell‐free extract (purity fold of 0.1). Notably, nitric oxide production by membrane proteins and cell‐free extract stopped after 4 min whereas the production by soluble proteins continued. Experiments on DDM‐solubilized membrane proteins also showed that activity ceased after 4 min (Fig. S1). Thus, the membranes themselves did not interfere with nitrite reductase activity, for example, by preventing membrane‐bound nitrite reductases from interacting with electron donors and carriers. The reason for the ceasing activity remains unknown. In conclusion, the majority of *K. stuttgartiensis* nitrite reductase activity could be attributed to the soluble protein fraction, indicating that the active nitrite reductase is soluble.

First, the total activity of each fraction was determined and compared to the total activity of the starting material, to identify the fractions containing active nitrite reductase. Subsequently, to identify the active nitrite reductases among the soluble proteins, we enriched nitrite reductases via FPLC. Active nitrite reductases were identified in the different protein fractions via enzyme activity assays, in which reduction of ^15^N‐labeled nitrite to ^15^N‐nitric oxide was followed. Subsequently, combining the measured specific activities with proteomics analysis allowed for the identification of the active nitrite reductase per protein fraction.

### Initial separation of soluble proteins via low‐resolution FPLC

**Fig. 2:**
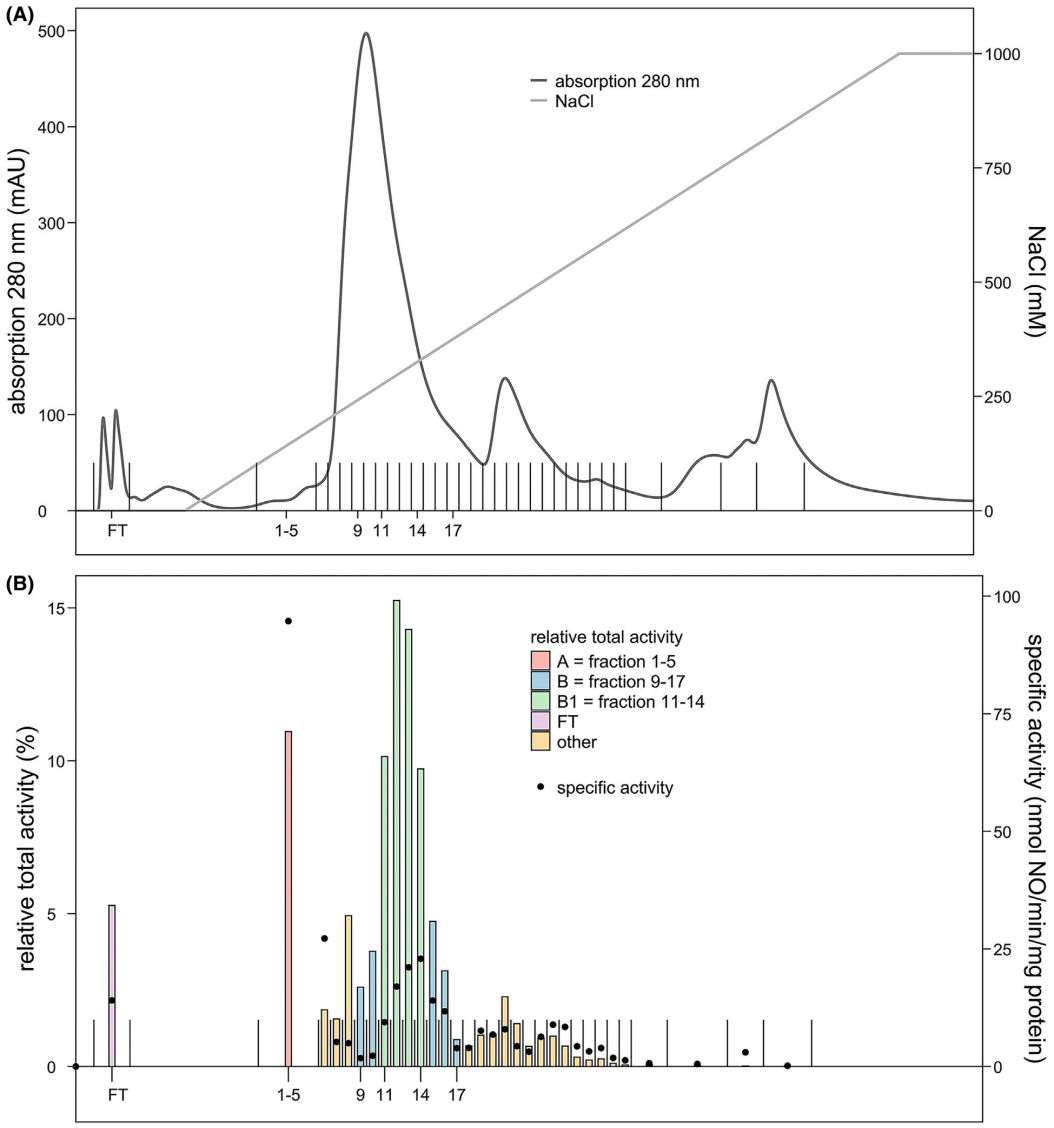
Initial separation of *Kuenenia stuttgartiensis* soluble proteins with nitrite reductase activity. (A) For the initial separation of soluble proteins, the proteins were separated on a low‐resolution anion‐exchange column with a linear gradient from 0 to 1 m NaCl. Eluted proteins were collected in 10 mL fractions. When the 280 nm UV signal was ≤ 25 mAU and thus indicated a low protein concentration, the fractions were pooled. (B) Of the total nitrite reductase activity of soluble proteins, 65% was measured in fractions 9–17, 50% was measured in fractions 11–14, 11% was measured in pooled fraction 1–5, and 5.3% was measured in the flow‐through (FT, nonbinding proteins). The majority of nitrite reductase activity was present in a single chromatographic peak of separated *K. stuttgartiensis* soluble proteins. Activity assays contained 200 μm ascorbate and phenazine ethosulfate, and 6–10 μg protein in 20 mm MOPS, 150 mm NaCl buffer, pH 7.5. The reaction was started with 77 μm ^15^N‐nitrite and carried out at 30 °C. The relative total activity compared to activity of the total soluble proteins is expressed in percentage. The specific activity is indicated by the black dots (*n* = 1).

For their initial separation, soluble proteins were fractionated with a low‐resolution anion‐exchange column (Fig. 2). Nitrite reductase activity in the column fractions was measured with MIMS and showed that protein fractions 9–17 formed a peak in total nitrite reductase activity with the highest specific rate of 23 nmol nitric oxide·min^−1^·mg^−1^ protein in fraction 14 (Fig. 2—note that the black dots display the specific activity in Fig. 2B). Together, the nine fractions accounted for 65% of nitrite reductase activity measured in the soluble proteins and had a specific activity of 12 nmol nitric oxide·min^−1^·mg^−1^ protein (for an overview see Table S1 and Fig. 8). For clarity, the pooled fractions 9–17 were renamed to sample B. The most active fractions 11–14 within sample B were renamed to sample B1 and have a specific activity rate of 18 nmol nitric oxide·min^−1^·mg^−1^ protein and account for 49% of the total nitrite reductase activity measured in all soluble proteins. The second fraction that contributed most to the total nitrite reductase activity of soluble proteins was pooled fraction 1–5. Here, nitric oxide was produced at 95 nmol·min^−1^·mg^−1^ protein and the sample accounted for 11% of the total nitrite reductase activity in soluble proteins. The pooled fractions 1–5 were renamed ‘sample A’. The flow‐through (FT), containing proteins that did not bind to the column, was the third fraction in terms of relative total activity. Here, nitrite reductase produced 14 nmol nitric oxide·min^−1^·mg^−1^ protein which accounted for 5.3% of the total nitrite reductase activity measured in all soluble proteins. To identify the active nitrite reductase(s) in *K. stuttgartiensis*, the samples FT, A, B, and B1 were further investigated.

### Enrichment and identification of active, soluble nitrite reductase

#### High‐resolution separation of sample B enriched nitrite reductase while preserving activity

The peak of nitrite reductase activity appeared in the second half of the UV absorption peak of sample B, suggesting that nitrite reductase is present in this subsection of the major UV peak (Fig. 2). To further enrich nitrite reductase in sample B, we used high‐resolution anion‐exchange column chromatography for enhanced separation and resolution. First, soluble proteins were again separated on a low‐resolution anion‐exchange column to obtain sample B. The sample accounted for 85% of the total nitrite reductase activity in soluble proteins (for an overview see Table S1 and Fig. 8). Next, the proteins of sample B were loaded on a high‐resolution anion‐exchange column and eluted via a linear gradient from 0 to 1 m NaCl. This resulted in four UV peaks in which nitrite reductase activity was measured (Fig. S2). In UV peak 4, nitrite reductase was most active and produced 36 nmol nitric oxide·min^−1^·mg^−1^ protein. Furthermore, the enzyme was 5.7 times enriched compared to the soluble proteins. The proteins in UV peak 4 accounted for 60% of the total nitrite reductase activity among the soluble proteins (Fig. S2 and for an overview see Table S1 and Fig. 8). In conclusion, the two‐step approach combining low‐ and high‐resolution anion‐exchange chromatography enriched nitrite reductase while largely preserving its activity.

#### High‐resolution separation of sample B1 enriched NirS

Next, we optimized the two‐step enrichment procedure to further isolate the active nitrite reductase in sample B. Almost all nitrite reductase activity in sample B originated from fractions 11–14 (Fig. 2). Thus, soluble proteins were separated on a low‐resolution anion‐exchange column a third time, and only fractions 11–14 were pooled to obtain sample B1. Now the sample had a specific activity of 9.7 nmol nitric oxide·min^−1^·mg^−1^ protein and accounted for 22% of the total nitrite reductase activity in soluble proteins, instead of the expected rate of 18 nmol nitric oxide·min^−1^·mg^−1^ protein and production of 49% that was measured in the first low‐resolution separation (for an overview see Table S1 and Fig. 8). Possibly, variation in elution patterns resulted in differences in total activities measured. Alternatively, nitrite reductase may lack essential accessory components, such as cofactors or stabilizing proteins, following enrichment. However, such proteins could not be detected (Table S2). Nevertheless, separation of soluble proteins seems to lead to a loss in enzyme activity.

**Fig. 3:**
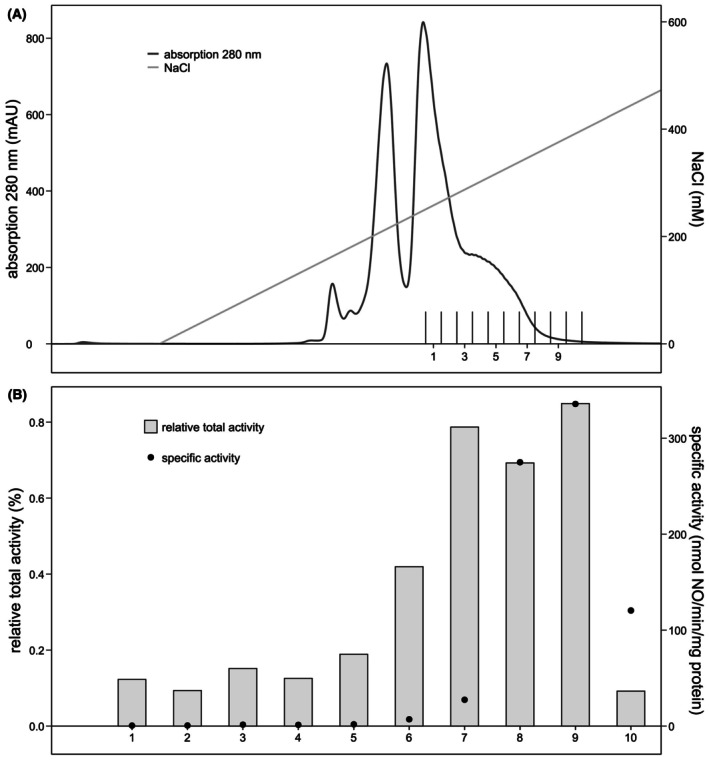
Separation of the *Kuenenia stuttgartiensis* proteins in sample B1 by high‐resolution anion‐exchange column chromatography and their nitrite reductase activity. (A) Soluble proteins were first separated on a low‐resolution anion‐exchange column of which sample B1 showed high‐specific and relative total activity compared to activity measured in all soluble proteins. To enrich the active nitrite reductase in sample B1, proteins were further separated on a high‐resolution anion‐exchange column. Proteins were eluted with a linear gradient from 0 to 1 m NaCl in 1 mL fractions. (B) Fraction 9 of the high‐resolution anion‐exchange column was the most enriched and most active fraction after two‐step column chromatography separation. Here, nitrite reductase produced 336 nmol nitric oxide·min^−1^·mg^−1^ protein which accounted for 0.8% of the total nitrite reductase activity measured for all soluble proteins. Activity assays contained 200 μm ascorbate and phenazine ethosulfate, and 6–10 μg protein in 20 mm MOPS, 150 mm NaCl buffer, pH 7.5. The reaction was started with 77 μm ^15^N‐nitrite and carried out at 30 °C. The relative total activity compared to activity of the total soluble proteins is expressed in percentage. The specific activity is indicated by the black dots (*n* = 1).

To further enrich active nitrite reductase in sample B1, the proteins in this sample were separated on the high‐resolution anion‐exchange column (Fig. 3). This time, the eluted proteins were collected in 1 mL fractions independent of the UV signal. Of these collected fractions, nitric oxide was produced at 336 nmol·min^−1^·mg^−1^ protein in fraction 9, and nitrite reductase was 29 times enriched compared to the soluble proteins. This most active fraction accounted for 0.8% of the total nitrite reductase activity measured in the soluble protein fraction (Figs 3 and 8, Table S1). Notably, the relative total activity of the high‐resolution fractions 1–10 only attributed for 3.5% to the total nitrite reductase activity of soluble proteins. It cannot be ruled out that proteins that eluted prior to fractions 1–10 also produced nitric oxide and that the percentage of total nitrite reductase activity was higher than 3.5%.

**Fig. 4:**
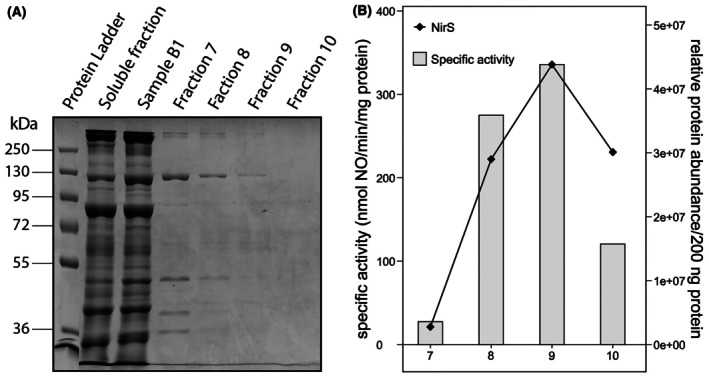
SDS/PAGE of *Kuenenia stuttgartiensis* soluble proteins and sample B1 proteins separated via high‐resolution anion‐exchange column chromatography, and correlation between the relative abundance of NirS and the nitrite reductase activity of these proteins. (A) SDS/PAGE of the soluble fraction, sample B1, and fractions 7, 8, 9, and 10 that showed nitrite reductase activity after separation with high‐resolution anion‐exchange column chromatography. The protein bands in the fractions obtained with high‐resolution anion‐exchange chromatography are barely visible due to the low protein concentration in the fractions. Of the soluble protein fraction and sample B1, 42 μg protein was loaded on a 10% SDS/PAGE gel. Of fractions 7, 8, 9, and 10 obtained with high‐resolution anion‐exchange chromatography, 5, 13, 13, and 12 μg protein was loaded on a 10% SDS/PAGE gel, respectively. (B) The relative abundance of NirS correlated well with the specific nitrite reductase activity measured in the high‐resolution fractions (the specific activities are also indicated as black dots in Fig. 3B) (*n* = 1).

To evaluate the purity of protein fraction 9, proteins were loaded onto an SDS/PAGE gel. However, the low protein concentration in this fraction prevented detection of the proteins (Fig. 4A). To identify the active nitrite reductase in protein fraction 9, we subjected protein fractions 7–10 to proteomics analysis and surveyed the correlation between the relative abundance of identified nitrite reductase and specific activity per fraction. Proteomics revealed that NirS had the highest relative abundance measured in protein fraction 9 (Table S3). Moreover, Pearson correlation analysis showed the highest positive correlation between the relative abundance of all detected proteins and specific nitric oxide‐producing activity for NirS (*r* = 0.87) (Fig. 4B, Table S3). Other proteins with a relative abundance that positively correlated with the specific activities measured per fraction are not known to have nitrite reductase capabilities (though it cannot be fully excluded that there is an as‐yet‐unidentified nitrite reductase among them). These results strongly suggest that NirS is the enzyme responsible for the nitrite reductase activity in sample B.

#### The nitrite reductase in sample A remains unknown

**Fig. 5:**
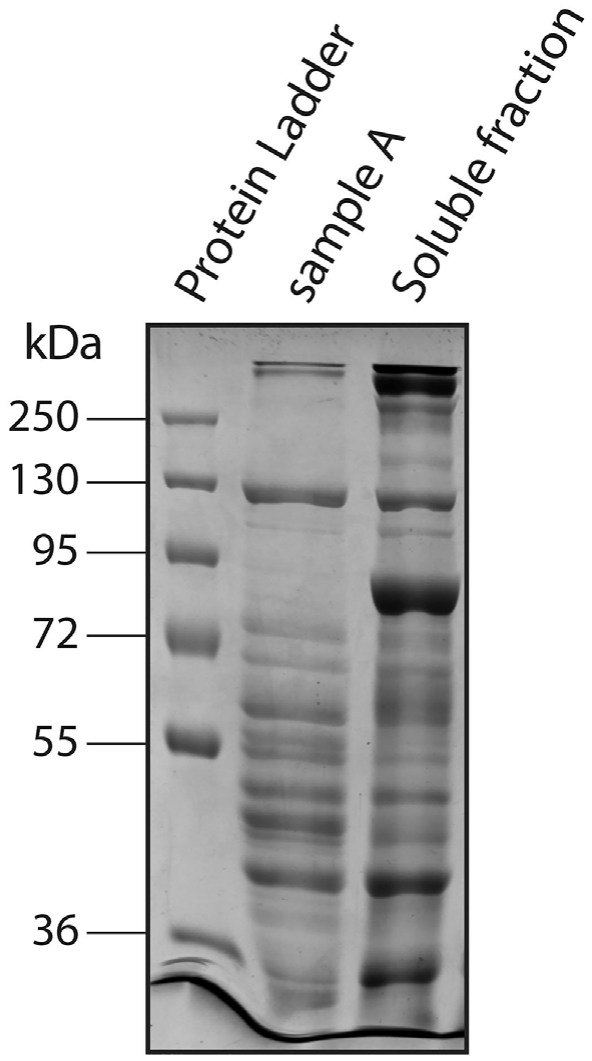
SDS/PAGE of the *Kuenenia stuttgartiensis* soluble protein fraction and sample A obtained with low‐resolution anion‐exchange column chromatography. The number of bands on the gel indicated that sample A contained multiple proteins. Of both samples, 42 μg protein was loaded on a 10% SDS/PAGE gel.

Low‐resolution sample A accounted for 11% of the total nitrite reductase activity among soluble proteins (Fig. 2 and for an overview see Table S1 and Fig. 8). SDS/PAGE showed that the sample contained various proteins (Fig. 5). Despite the high number of different proteins present in this fraction, the calculated purity fold of nitrite reductase in sample A compared to total soluble proteins was surprisingly high (12‐fold). Proteomics analysis did not reveal any known or potential nitrite reductases (Table S4), probably because the sample was too complex and had a low protein concentration.

#### High‐resolution separation of FT proteins enriched HAOr

**Fig. 6:**
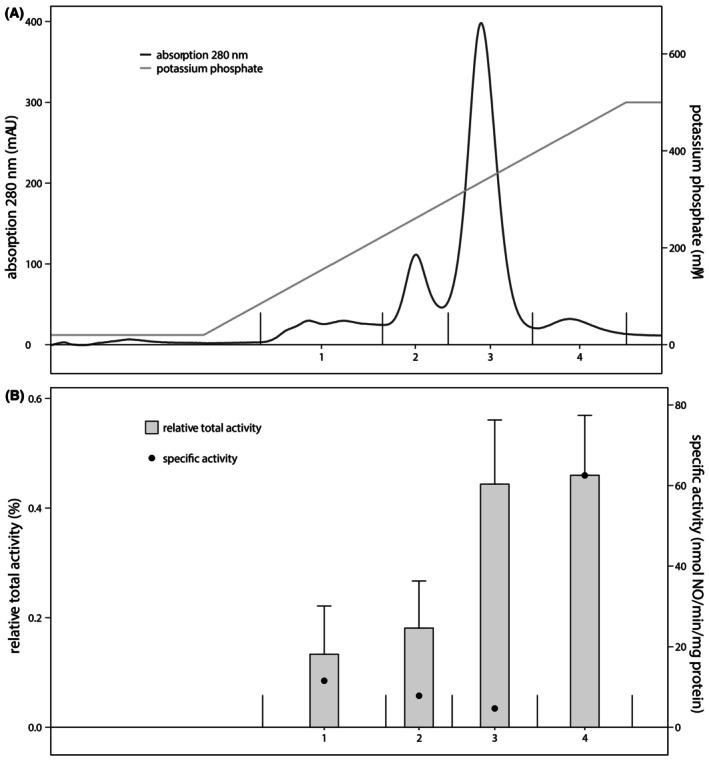
Separation of the *Kuenenia stuttgartiensis* proteins in sample FT by mixed‐mode column chromatography and their nitrite reductase activity. (A) FT proteins collected from low‐resolution anion‐exchange column chromatography were further fractionated via mixed‐mode chromatography with a Hydroxyapatite column with a linear gradient from 20 to 500 mm potassium phosphate buffer. Proteins eluted in four distinguished UV peaks. (B) Most nitrite reductase activity in FT proteins was measured in UV peak 4 of the mixed‐mode chromatography column. The nitrite reductase activity accounted for 0.5% of the total nitrite reductase activity measured for all soluble proteins. Activity assays contained 200 μm ascorbate and phenazine ethosulfate, and 6–10 μg protein in 20 mm MOPS, 150 mm NaCl buffer, pH 7.5. The reaction was started with 77 μm ^15^N‐nitrite and carried out at 30 °C. The relative total activity compared to activity of the total soluble proteins is expressed in percentage. The specific activity is indicated by the black dots. Data represented as the mean ± SD (*n* = 4 biological replicates).

**Fig. 7:**
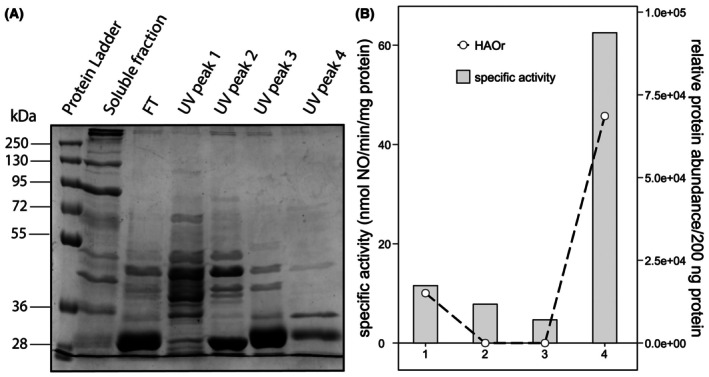
SDS/PAGE of *Kuenenia stuttgartiensis* soluble proteins and FT proteins separated via mixed‐mode column chromatography, and correlation between the relative abundance of HAOr and the nitrite reductase activity of these proteins. (A) SDS/PAGE characterization of FT proteins obtained via low‐resolution anion‐exchange chromatography and proteins obtained via mixed‐mode column chromatography. UV peak 4 showed the lowest number of visible bands. For all samples, 20 μg protein was loaded on a 10% SDS gel. (B) The relative abundance of HAOr per 200 ng protein and the specific activity in nmol nitric oxide per min per mg protein measured per fraction (the specific activities are also indicated as black dots in Fig. 6B). The relative abundance of HAOr correlated well with the nitrite reductase activity (*n* = 1).

**Fig. 8:**
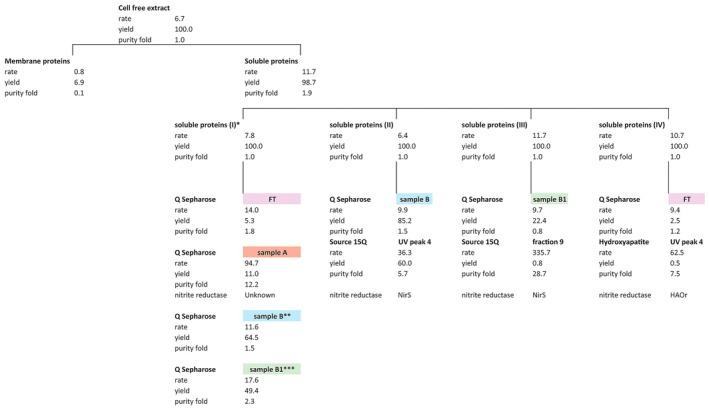
Overview of the enrichment of active nitrite reductases in *Kuenenia stuttgartiensis*. The majority of the nitrite reductase activity was measured in the soluble proteins. To identify the active nitrite reductase, soluble proteins were separated with fast protein liquid chromatography. Nitrite reductase activity per fraction was followed over time via the ^15^N‐nitric oxide production from ^15^N‐nitrite. Activity assays contained 200 μm ascorbate and phenazine ethosulfate, 6–10 μg protein in 20 mm MOPS, 150 mm NaCl buffer, pH 7.5. The reaction was started with 77 μm ^15^N‐nitrite and carried out at 30 °C. The activity assays showed that FT, sample A, and sample B contributed most to the nitrite reductase activity. With proteomics, we identified HAOr and NirS as the active nitrite reductases. The rate is expressed in nmol nitric oxide·min^−1^·mg^−1^ protein, yield in % activity relative to total activity measured in the cell extract or soluble proteins, and purity fold is the specific activity per fraction compared to specific activity measured in cell extract or soluble proteins. *Roman number indicates the replicate of soluble proteins from which proteins are further separated with fast protein liquid chromatography. **For sample B, values of fractions 9–17 were combined. For the specific activity and concentration, the average of fractions 9–17 was used. For the other measurements, the sum of the individual fractions was used. ***For sample B1, values of fractions 11–14 were combined. For the specific activity and concentration, the average of fractions 11–14 was used. For the other measurements, the sum of the individual fractions was used.

The third protein fraction that contributed most to the total nitrite reductase activity was sample FT from the low‐resolution anion‐exchange column (Fig. 2 and for an overview see Table S1 and Fig. 8). To identify the active nitrite reductase in FT, the proteins were further separated via mixed‐mode chromatography on a ceramic hydroxyapatite column. A linear gradient of 20–500 mm potassium phosphate resulted in an elution pattern with four UV peaks in which nitric oxide production from nitrite was measured (Fig. 6 and for an overview see Table S1 and Fig. 8). Although the activities differed between biological replicates, ratios between the total activities of the UV peaks within each replica were consistent: Nitrite reductase activity in UV peaks 1 and 2 was 2.5‐ to 3‐fold lower than activity measured in UV peaks 3 and 4 (Fig. 6). The highest specific activity was measured in UV peak 4, in which nitric oxide production proceeded at on average 62.5 nmol·min^−1^·mg^−1^ protein. In accordance with the low number of visible bands on the SDS/PAGE gel, the enrichment of nitrite reductase was highest in this fraction: 7.5 ± 4 times compared to all soluble proteins (Fig. 7A and for an overview see Table S1 and Fig. 8).

Proteome analysis on fractions corresponding to UV peaks 1 to 4 identified the nitrite reductases NirS and HAOr. Pearson correlation analysis between specific nitrite reductase activity and the relative abundance of NirS and HAOr showed a strong correlation for HAOr (*r* = 0.99) (Fig. 7B) and a weak correlation for NirS (*r* = −0.19) (Table S5). Other proteins with a relative abundance that positively correlated with the specific activities measured per fraction are not known to function as nitrite reductase (though it cannot be fully excluded that there is an as‐yet‐unidentified nitrite reductase among them). Therefore, HAOr is presumably the active nitrite reductase in this protein fraction.

## Discussion

In this study, we identified active nitrite reductases in *K. stuttgartiensis* strain MBR1 to gain more insight in the puzzling redundancy of this enzyme in anammox bacteria. First, we showed that the active nitrite reductases are soluble proteins. In the soluble protein fraction, nitrite reductase is two times enriched compared to the cell‐free extract and almost all nitrite reductase activity in the cell‐free extract is recovered in the soluble protein fraction (> 98%). On the contrary, activity in the membrane‐bound proteins is minor and stopped shortly after the start of the assays. Using activity‐guided enrichment of soluble proteins and proteomics, we identified NirS and HAOr as active nitrite reductases in two dominant activity peaks detected after low‐resolution anion‐exchange chromatography (65% of the total nitrite reductase activity in sample B and 5.3% in FT). Interestingly, the enriched nitrite reductases have different nitrite reduction rates. Next, we will further discuss the role of the two soluble nitrite reductases in the anammox metabolism.

### NirS

NirS is one of the active soluble nitrite reductases in *K. stuttgartiensis* identified in this study. In this anammox species, NirS is a low abundant protein compared to the other enzymes central in the anammox catabolism, that is, hydrazine synthase and hydrazine dehydrogenase [27, 36, 39]. It could be speculated that NirS has a high‐specific activity rate to meet the nitric oxide demands of hydrazine synthase. Here, we measured that the most active fraction, fraction 9 of the low‐resolution anion‐exchange column, contained NirS and produced 336 nmol nitric oxide·min^−1^·mg^−1^ protein. In previous research, *K. stuttgartiensis* batch cultures grown on nitrite and ammonium produced 30 nmol dinitrogen gas·min^−1^·mg^−1^ protein [10]. Based on the measured rate in our study, NirS would need to constitute 10% of the total protein content of the cell to produce sufficient nitric oxide, which contradicts its low abundance at the proteome level. The measured rate may be underestimated due to the presence of other proteins as NirS was not purified to homogeneity in this fraction 9. Also, in a comparative study, NirS has been shown to form a highly active complex with its native electron donor, but low activity with chemical redox mediators [51]. In denitrifiers, NirS produces up to 4.15 μmol nitric oxide·min^−1^·mg^−1^ protein [37], about 12 times faster than our rate measured for enriched *K. stuttgartiensis* NirS. The denitrifier rate aligns more closely with the NirS levels that could be expected in anammox cells based on transcriptome and proteome data. Further isolation of *K. stuttgartiensis* NirS could be a next step to determine whether its activity rate can be further increased compared to the value measured in this study.

### HAOr

In addition to NirS, we identified HAOr as one of the active nitrite reductases in *K. stuttgartiensis*. The most active fraction in sample FT of the low‐resolution anion‐exchange column produced 62.5 ± 30 nmol nitric oxide·min^−1^·mg^−1^ protein. The activity in this sample could be attributed to HAOr. This would indicate that the catalytic activity of enriched HAOr is 125 times higher than the activity measured for the purified *K. stuttgartiensis* HAOr [9] in a nearly identical assay setup. The difference in activity is possibly caused by the isolation procedure. While Ferousi *et al*. [9] eluted HAOr isocratically with 550 mm NaCl from a Q‐Sepharose column, we enriched the protein from the FT of the same type of column. In fact, we were unable to identify HAOr in the proteins bound to the low‐resolution anion‐exchange column in our experiments. This may be due to impurities in the fractions. Isolating HAOr from the same low‐resolution anion‐exchange fractions as Ferousi *et al*. [9], rather than the FT, would be valuable to avoid potential interference from proteins like NirS, which was identified in the FT as well, for enzyme activity determination. Further isolation of the nitrite reductase in active protein fractions bound to this column could be a next step.

### Possible role of multiple nitrite reductases in anammox bacteria

This study demonstrates that *K. stuttgartiensis* contains two active soluble nitrite reductases, consistent with prior research showing that the *nirS* and *haor* genes are both differentially expressed under environmental nitrite limitation [27, 40]. The redundancy in nitrite reductases is not a phenomenon unique to anammox bacteria. Some denitrifiers express both *nirK* and *nirS* genes, but the functional advantage of this dual expression is not fully understood [52, 53, 54]. For instance, it has not yet been demonstrated that NirS and NirK are both functional when present in the genome of the same denitrifier nor what mechanisms determine which nitrite reductase is expressed [52].

In *K. stuttgartiensis*, HAOr and NirS could be differentially expressed under distinct conditions aiding in the flexibility of the anammox bacteria to adapt to various environmental conditions. For instance, under low nitrite conditions it could be beneficial to express high‐affinity nitrite reductase to maintain growth. It would be interesting to study whether HAOr and NirS differ in nitrite affinity. NirS is a nitrite reductase with a high *K*_m_ between 6 and 53 μm nitrite in denitrifiers [37]. However, nitrite uptake is likely the constraining factor in nitrite‐limited conditions and not the nitrite reductase rate.

In addition to improving adaptability of *K. stuttgartiensis* to environmental changes, the various anammox nitrite reductases could also be distributed to different parts of the cell. In denitrifying bacteria, nitrite reduction to nitrous oxide is coupled to energy conservation via oxidative phosphorylation [55]. Consequently, nitrite reductase is located close to the cytoplasmic membrane and mostly in the periplasm [56]. In anammox bacteria, energy is proposed to be conserved over the anammoxosome membrane [5]. Nitrite reduction to nitric oxide requires electrons that are probably donated by one of the Rieske/cytochrome *b* complexes of anammox bacteria [15] or the anammox nitrite oxidoreductase (NXR) [57]. Immunogold labeling studies confirmed that HAOr is located in the vicinity of the anammoxosome membrane [38]. However, the localization of NirS has not been determined yet. To understand why anammox bacteria harbor various nitrite reductases, it would be valuable to locate these enzymes in different anammox bacteria and under varying growth conditions.

While preparing this manuscript for submission, a publication [58] came out where nitrite‐reducing activity was followed in *K. stuttgartiensis* strain CSTR1 [59] using several fractionation methods. The *in vitro* assay detected nitrite‐reducing activity in *K. stuttgartiensis* CSTR1 cell extracts and protein fractions and confirmed nitric oxide as the product. Their size exclusion chromatography data suggested the hydroxylamine dehydrogenase (HOX; kustc1061, GenBank: SOH05157.1) to play a role as nitrite reductase; however, these results could not be verified by the anion‐exchange chromatography experiments. While HOX can be forced to work as a reductase *in vitro* through the use of strong reductants such as methyl viologen, it seems to be tuned towards oxidative catalysis [60]. Its P460 cofactor remains oxidized in the presence of weaker reductants, thus strongly favoring oxidative reactions [60], making an *in vivo* role in nitrite reduction unlikely. Here, we detected HOX in protein fraction B1 (Fig. 8) but not in the other fractions. In fraction B1, HOX had a lower Pearson correlation score (*r*) between specific activity and relative protein abundance compared to NirS: HOX *r* = 0.85 (compared to *r* = 0.92 for NirS) and after further separation on a high‐resolution anion‐exchange column HOX *r* = 0.60 (compared to *r* = 0.87 for NirS) (Table S2—line 9, and Table S3—line 10). In our *K. stuttgartiensis* strain MBR1 and data, NirS is thus identified as the main contributor to nitric oxide production in sample B1. Ude *et al*. [58] did not find evidence for either NirS or HAOr to be involved in nitrite reduction in their experiments and *K. stuttgartiensis* strain. In their paper, Ude *et al*. [58] conclude that multiple proteins may be involved in nitrite reduction underpinning our findings that there seems to be no single dedicated nitrite reductase in anammox bacteria in general or species/strains specifically.

## Conclusion

We have shown that *K. stuttgartiensis* strain MBR1 contains at least two nitric oxide‐forming nitrite reductases, NirS and HAOr, that are active *in vitro*. This suggests that the anammox bacterium employs two nitrite reductases to keep its nitric oxide pool replenished. Containing two different nitrite reductases could improve versatility of *K. stuttgartiensis* to changes in environmental nitrite concentrations. For instance, because the enzymes have different rates and affinities. Furthermore, the different nitrite reductases could be located in different parts of the cell. While we could identify nitrite reductases that are present and active in *K. stuttgartiensis*, results of enzyme assays are difficult to translate to physiological enzyme activity. To follow up on this study, it would be interesting to evaluate nitrite reductase activity in different anammox species and within species grown in different nitrite concentrations. Moreover, the active protein fractions isolated here could be further enriched in nitrite reductase to identify and characterize the enzyme present there. Lastly, immunogold localization could enhance the understanding of nitrite reductase location and function within the cell. In conclusion, the redundancy in genes encoding potential nitrite reductases, along with the identification and activity of two distinct enzymes, underscores the critical role of nitric oxide in anammox bacteria.

## Conflict of interest

The authors declare no conflict of interest.

## Author contributions

LN and WV conceptualized and supervised the project; LN obtained funding; all authors designed experiments; FJV, LWN, and WV performed experiments; all authors analyzed data; FJV and LWN wrote the first draft of the manuscript; and all authors reviewed, edited, and approved the manuscript.

## Supporting information


**Fig. S1.** ^15^N‐labeled nitric oxide production from ^15^N‐labeled nitrite by cell‐free anammox extract, soluble protein fraction, and membrane protein fraction incubated with 1% *n*‐dodecyl β‐d‐maltoside (DDM).

**Fig. S2.** Separation of the proteins in sample B by high‐resolution anion‐exchange column chromatography and their nitrite reductase activity.

**Table S1.** Overview of the enrichment of active nitrite reductases in *K. stuttgartiensis*.

**Table S2.** Proteins identified in sample B1 (low‐resolution fractions 11–14) and low‐resolution fractions 9 and 10.

**Table S3.** Correlation between the relative abundance of identified proteins and specific nitrite reductase activity in the fractions obtained from sample B1 that were further separated on a high‐resolution anion‐exchanger.

**Table S4.** Correlation between the relative abundance of identified proteins and specific nitrite reductase activity in three samples obtained with low‐resolution column chromatography: FT, sample A, and low‐resolution fraction 6.

**Table S5.** Correlation between the relative abundance of identified proteins and specific nitrite reductase activity in all fractions obtained with mixed‐mode column chromatography.

## Data Availability

The data that support the findings of this study are available in the Supporting Information of this article. In addition, biological samples used in this study are available from the corresponding author (laura.vanniftrik@ru.nl) upon reasonable request.
