# Supplementary material for: Nitric oxide‐forming nitrite reductases in the anaerobic ammonium oxidizer Kuenenia stuttgartiensis
Source: FEBS Open Bio. 2025 Aug 4;15(10):1696–713. doi: 10.1002/2211-5463.70086 (PMC12485887; doi:10.1002/2211-5463.70086)
Supplement: Supplementary file 1 — Fig. S1. 15N‐labeled nitric oxide production from 15N‐labeled nitrite by cell‐free anammox extract, soluble protein fraction, and membrane protein fraction incubated with 1% n‐dodecyl β‐d‐maltoside (DDM). [file FEB4-15-1696-s005.pdf]

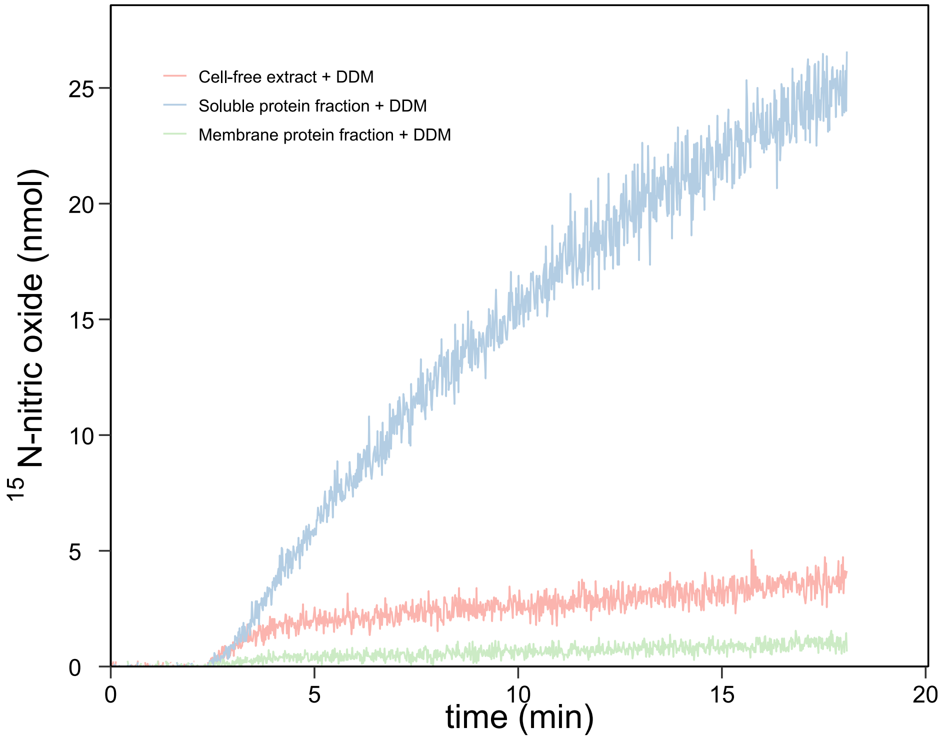

Supplementary figure 1 – ^{15}N-labeled nitric oxide production from ^{15}N-labeled nitrite by cell-free anammox extract, soluble protein fraction, and membrane protein fraction incubated with 1% n-dodecyl β-D-maltoside (DDM). Nitric oxide production in membrane proteins and cell-free extract stopped around four minutes whereas the production in soluble proteins continued. Activity assays contained 200 µM ascorbate and phenazine ethosulfate, and 6-10 µg protein in 20 mM MOPS, 150 mM NaCl buffer, pH 7.5. The reaction was started with 77 µM ^{15}N-nitrite and carried out at 30°C. (*n*= 1)
