# Supplementary material for: Nitric oxide‐forming nitrite reductases in the anaerobic ammonium oxidizer Kuenenia stuttgartiensis
Source: FEBS Open Bio. 2025 Aug 4;15(10):1696–713. doi: 10.1002/2211-5463.70086 (PMC12485887; doi:10.1002/2211-5463.70086)
Supplement: Supplementary file 2 — Fig. S2. Separation of the proteins in sample B by high‐resolution anion‐exchange column chromatography and their nitrite reductase activity. [file FEB4-15-1696-s001.pdf]

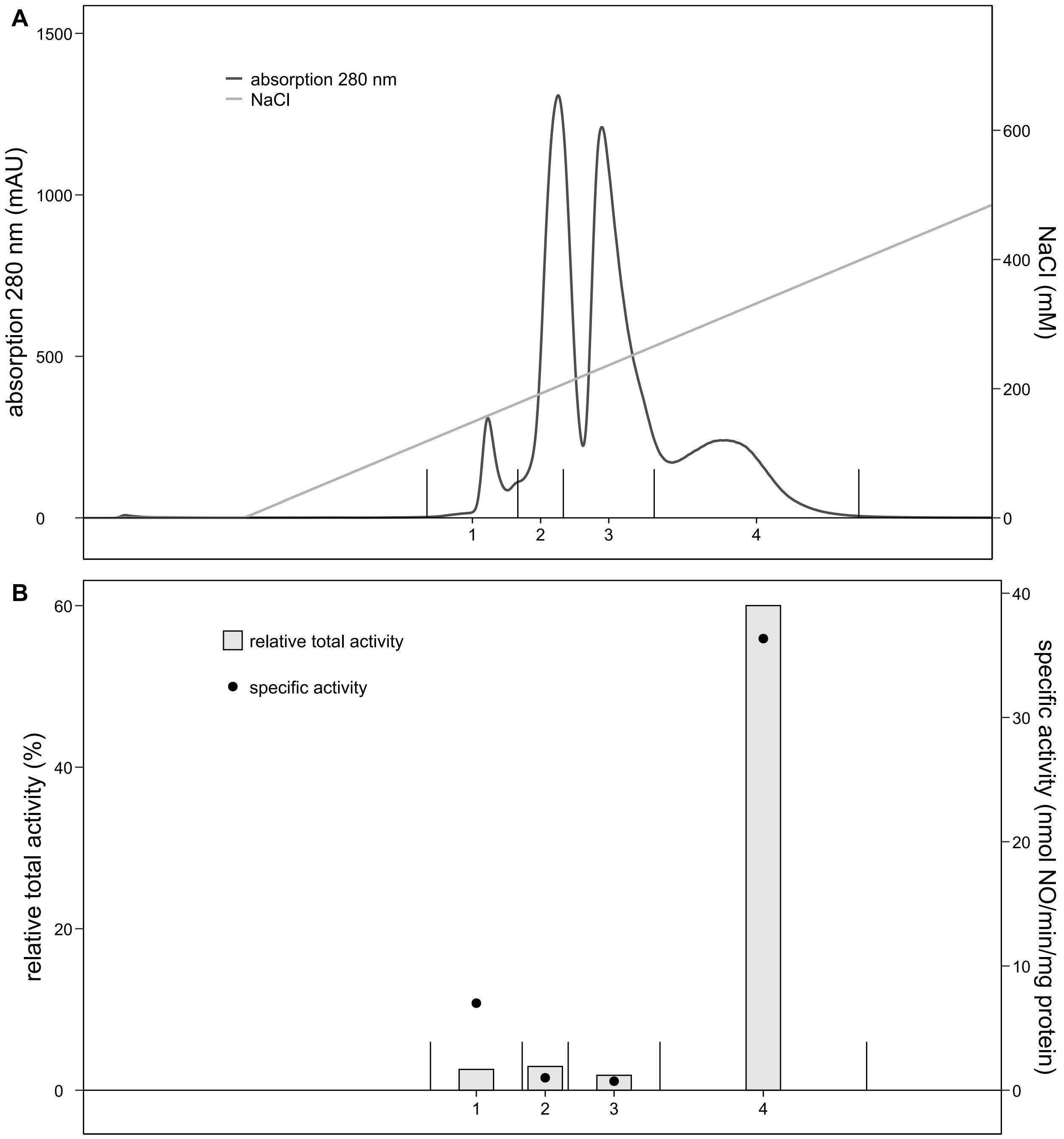

Supplementary figure 2 – Separation of the proteins in sample B by high-resolution anion-exchange column chromatography and their nitrite reductase activity. (A) Soluble proteins were initially separated on a low-resolution column of which sample B showed high specific and relative total activity compared to activity measured in all soluble proteins. To enrich the active nitrite reductase in sample B, proteins were further separated on a high-resolution anion-exchanger. Proteins were eluted with a linear gradient from 0 to 1 M NaCl and fractionated based on their UV signal. (B) UV peak 4 was the most enriched and most active fraction after two-step column chromatography separation. Here, nitrite reductase produced 36 nmol nitric oxide/min/mg protein which accounted for 60% of the total nitrite reductase activity measured for all soluble proteins. Activity assays contained 200 µM ascorbate and phenazine ethosulfate, 6-10 µg protein in 20 mM MOPS. 150 mM NaCl buffer, pH 7.5. The reaction was started with 77 µM ^{15}N-nitrite and carried out at 30°C. The relative total activity compared to activity in the total soluble protein fraction is expressed in percentage. The specific activity is indicated by the black dots. (*n*=1)
