# Supplementary material for: Nitric oxide‐forming nitrite reductases in the anaerobic ammonium oxidizer Kuenenia stuttgartiensis
Source: FEBS Open Bio. 2025 Aug 4;15(10):1696–713. doi: 10.1002/2211-5463.70086 (PMC12485887; doi:10.1002/2211-5463.70086)
Supplement: Supplementary file 3 — Table S1. Overview of the enrichment of active nitrite reductases in K. stuttgartiensis. [file FEB4-15-1696-s002.pdf]

Supplementary table 1 – Overview of the enrichment of active nitrite reductases in *K. stuttgartiensis*. The majority of the nitrite reductase activity was measured in the soluble protein fraction. To identify the active nitrite reductase, soluble proteins were separated with fast protein liquid chromatography. Nitrite reductase activity per fraction was followed over time via the ^{15}N-nitric oxide production from ^{15}N-nitrite. Activity assays contained 200 µM ascorbate and phenazine ethosulfate, 6-10 µg protein in 20 mM MOPS, 150 mM NaCl buffer, pH 7.5. The reaction was started with 77 µM ^{15}N-labeled nitrite and carried out at 30°C. The activity assays revealed that FT, sample A, and sample B contributed most to the nitrite reductase activity. With proteomics we identified HAOr and NirS as the active nitrite reductases.

column	sample	[protein] (mg/mL)	volume (mL)	total protein (mg)	specific activity (nmol/min/mg protein)	total activity (nmol/min)	yield (%)	purity fold	nitrite reductase
-	cell free extract	14.1	13.9	196.44	6.7	1319.2	100.0	1.0	
-	soluble proteins	7.1	5.8	39.50	11.7	463.7	98.7	1.9	
-	membrane proteins	5.8	6.5	49.40	0.8	39.0	6.9	0.1	
-	soluble proteins (I)*	22.8	10.00	227.50	7.8	1773.3	100.0	1.0	
Q Sepharose	FT	14.8	0.45	6.65	14.0	93.4	5.3	1.8	
Q Sepharose	sample A	3.4	0.60	2.05	94.7	194.2	11.0	12.2	Unidentified
Q Sepharose	sample B**	23.1	6.15	124.13	11.6	1.4	64.5	1.5	
Q Sepharose	sample B1***	23.4	2.70	54.5	17.6	1.0	49.4	2.3	
-	soluble proteins (II)	17.8	10.0	178.00	6.4	1140.0	100.0	1.0	
Q Sepharose	sample B	130.5	0.8	97.88	9.9	971.3	85.2	1.5	
Source 15Q	UV peak 1	2.6	1.6	4.20	7.0	29.4	2.6	1.1	
Source 15Q	UV peak 2	19.6	1.7	33.48	1.0	33.6	2.9	0.2	
Source 15Q	UV peak 3	18.2	1.6	28.94	0.7	21.1	1.9	0.1	
Source 15Q	UV peak 4	13.1	1.4	18.82	36.3	684.1	60.0	5.7	NirS
-	soluble proteins (III)	13.4	10.0	134.00	11.7	1572.1	100.0	1.0	
Q Sepharose	sample B1	48.6	0.8	36.45	9.7	352.0	22.4	0.8	
Source 15Q	Fraction 7	0.9	0.5	0.45	27.4	12.4	0.8	2.3	
Source 15Q	Fraction 8	0.1	0.5	0.04	275.0	10.9	0.7	23.5	
Source 15Q	Fraction 9	0.1	0.8	0.04	335.7	13.3	0.8	28.7	NirS
Source 15Q	Fraction 10	0.02	0.6	0.01	120.5	1.4	0.1	10.3	
-	soluble proteins (IV)	19.4	22.5	435.87	10.7	4673.2	100.0	1.0	
Q Sepharose	FT	2.1	5.0	12.59	9.4	118.7	2.5	1.2	
Hydroxyapatite	UV peak 1	0.8	0.6	0.55	11.6	6.4	0.1	1.0	
Hydroxyapatite	UV peak 2	1.6	0.6	1.17	7.8	9.1	0.2	0.9	
Hydroxyapatite	UV peak 3	7.3	0.6	4.81	4.7	22.5	0.5	0.5	
Hydroxyapatite	UV peak 4	0.5	0.6	0.35	62.5	21.9	0.5	7.5	HAOr

The rate in expressed nmol nitric oxide/min/mg protein, yield in % activity relative to total activity measured in the cell extract or soluble proteins, and purity fold is the specific activity per fraction compared to specific activity measured in cell extract or soluble proteins.

* Roman number indicates the replicate of soluble proteins from which proteins are further separated with fast protein liquid chromatography.

** For sample B values of fractions 9-17 were combined. For the specific activity and concentration the average of fractions 9-17 was used. For the other measurements the sum of the individual fractions was used.

*** For sample B1 values of fractions 11-14 were combined. For the specific activity and concentration the average of fractions 11-14 was used. For the other measurements the sum of the individual fractions was used.
