# Supplementary material for: Nitric oxide‐forming nitrite reductases in the anaerobic ammonium oxidizer Kuenenia stuttgartiensis
Source: FEBS Open Bio. 2025 Aug 4;15(10):1696–713. doi: 10.1002/2211-5463.70086 (PMC12485887; doi:10.1002/2211-5463.70086)
Supplement: Supplementary file 4 — Table S2. Proteins identified in sample B1 (low‐resolution fractions 11–14) and low‐resolution fractions 9 and 10. [file FEB4-15-1696-s004.pdf]

Supplementary table 2 - Proteins identified in sample B1 (low-resolution fractions 11-14) and low-resolution fractions 9 and 10. The positive Pearson correlation score calculated between specific activity measured per fraction and the relative protein abundance per fraction, showed that NirS is the only known active nitrite reductase contributing to nitric oxide production from nitrite in sample B. Other proteins that showed a positive correlation are not identified as nitrite reductases. Neither the low-resolution fractions 9 and 10 nor sample B1 contained known co-factors of NirS. Thus, the loss in nitrite reductase activity in sample B1 compared to sample B, is probably not due to missing co-factors. The data is ordered based on relative protein abundance that correlates best with the specific activity. Accession numbers refer to the *K. stuttgartiensis* protein sequence database in Uniprot (entry KSMBR1).

Protein number	Accession	Description	Relative protein abundance per fraction			Pearson correlation score between specific activity and relative protein abundance
			Low-resolution fraction 9	Low-resolution fraction 10	Sample B1	
1	Q1Q2D9	Putative periplasmic serine endoprotease DegP-like	2.50E+05	4.20E+05	5.94E+05	0.99
2	A0A2C9CDH8	Corrinoid/iron-sulfur protein large subunit	3.61E+05	3.29E+05	5.25E+05	0.97
3	Q1PYP7	RNA-binding protein	3.10E+05	4.03E+05	7.87E+05	0.94
4	Q1Q1P1	Putative succinyl-diaminopimelate desuccinylase	1.13E+04	3.92E+04	5.55E+04	0.94
5	A0A2C9CAZ0	Uncharacterized protein	1.81E+06	1.50E+06	2.02E+06	0.92
**6**	**Q1Q4F5**	**Strongly similar to cd1 nitrite reductase NirS**	**7.50E+05**	**7.28E+05**	**8.24E+05**	**0.92**
7	Q1PZK9	phphoribyl-AMP cyclohydrolase	2.11E+05	3.87E+05	2.98E+05	0.86
8	A0A2C9CBW4	CBS domain-containing protein	0.00E+00	1.73E+05	2.39E+05	0.85
9	Q1PX48	Hydroxylamine oxidoreductase , HOX	2.42E+07	2.42E+07	2.30E+07	0.85
10	Q1Q1F2	ATP phphoribyltransferase	0.00E+00	1.10E+05	1.49E+05	0.85
11	A0A2C9CJS7	Malate dehydrogenase	0.00E+00	7.89E+04	1.93E+05	0.85
12	A0A2C9CHI2	Ornithine carbamoyltransferase	0.00E+00	5.38E+04	6.65E+04	0.84
13	Q1Q6H8	Peptidyl-prolyl cis-trans isomerase C (Rotamase C)	7.23E+04	6.74E+04	7.30E+04	0.82
14	Q1Q4G9	Hemerythrin domain-containing protein	0.00E+00	1.89E+05	2.04E+05	0.82
15	A0A2C9CBU0	Sulfatase-modifying factor enzyme domain-containing protein	0.00E+00	1.70E+05	2.34E+05	0.82
16	Q1Q7H2	Ribe-phphate pyrophphokinase	2.33E+05	3.36E+05	2.13E+05	0.75
17	Q1PUK7	Formate--tetrahydrofolate ligase	5.23E+04	6.60E+04	2.56E+05	0.75
18	A0A2C9CJH3	NAD/GMP synthase domain-containing protein	1.77E+04	0.00E+00	5.53E+04	0.74
19	Q1Q6M7	FAD:protein FMN transferase	1.23E+04	0.00E+00	5.66E+04	0.74
20	A0A2C9CFH5	Aminotransferase class V domain-containing protein	1.55E+05	3.91E+04	1.78E+05	0.74
21	Q1Q2X4	3-oxoacyl-[acyl-carrier-protein] synthase 2	0.00E+00	8.18E+03	2.16E+05	0.73
22	A0A6G7GLF5	S-layer protein	1.42E+05	2.29E+05	1.54E+05	0.72
23	Q1Q7P4	Class IIb small soluble cyt c	7.10E+06	8.85E+06	5.53E+06	0.72
24	Q1Q2X1	Putative bacteriochlorophyllide d C-12(1)-methyltransferase	0.00E+00	0.00E+00	4.64E+04	0.72
25	Q1Q2W3	Carboxymuconolactone decarboxylase-like domain-containing protein	0.00E+00	0.00E+00	3.66E+04	0.72
26	Q1Q3H0	ATP synthase subunit beta	0.00E+00	0.00E+00	7.73E+05	0.72
27	A0A2C9CJI3	GatB/YqeY domain-containing protein	0.00E+00	0.00E+00	7.48E+04	0.72
28	Q1PW34	Uncharacterized protein	0.00E+00	0.00E+00	2.27E+04	0.72
29	A0A2C9CH96	Glutamine--tRNA ligase	0.00E+00	0.00E+00	1.88E+04	0.72
30	Q1Q201	Integration ht factor subunit beta	0.00E+00	0.00E+00	3.10E+04	0.72
31	Q1PZD7	Uncharacterized protein	0.00E+00	0.00E+00	1.00E+04	0.72
32	A0A2C9CEK8	D-3-phphoglycerate dehydrogenase	0.00E+00	0.00E+00	1.26E+04	0.72
33	Q1PVP3	3-oxoacyl-[acyl-carrier-protein] reductase FabG	0.00E+00	0.00E+00	1.04E+04	0.72
34	A0A2C9CH02	Bifunctional oligoribonuclease/PAP phphatase NrnA	0.00E+00	0.00E+00	6.22E+03	0.72
35	A0A2C9CIY9	Flagellar hook-length control protein-like C-terminal domain-containing protein	0.00E+00	0.00E+00	2.13E+04	0.72
36	A0A2C9CC45	Serine--tRNA ligase	0.00E+00	0.00E+00	1.01E+04	0.72
37	Q1Q3F2	SWIM-type domain-containing protein	0.00E+00	0.00E+00	6.22E+04	0.72
38	Q1Q5R5	Aspartokinase	0.00E+00	0.00E+00	1.77E+04	0.72
39	A0A2C9CGI1	Peroxiredoxin	0.00E+00	0.00E+00	8.01E+04	0.72
40	A0A2C9CFM5	Transcription termination factor Rho	0.00E+00	0.00E+00	7.44E+04	0.72
41	A0A2C9CJI9	site-specific DNA-methyltransferase (adenine-specific)	0.00E+00	0.00E+00	1.23E+05	0.72
42	Q1Q0M4	2-isopropylmalate synthase	0.00E+00	0.00E+00	2.89E+04	0.72
43	Q1Q045	12,18-didecarboxysiroheme deacetylase	0.00E+00	0.00E+00	3.29E+04	0.72
44	Q1PW15	ATP-dependent Clp protease proteolytic subunit	0.00E+00	0.00E+00	7.19E+04	0.72
45	A0A2C9CFE4	Lon protease	0.00E+00	0.00E+00	1.49E+05	0.72
46	Q1PVQ6	Uncharacterized protein	0.00E+00	0.00E+00	2.33E+04	0.72
47	A0A2C9CI21	Aminotransferase	0.00E+00	0.00E+00	3.21E+04	0.72
48	A0A2C9CH82	Flavodoxin-like domain-containing protein	0.00E+00	0.00E+00	1.19E+04	0.72

Protein number	Accession	Description	Relative protein abundance per fraction Low-resolution fraction 9	Low-resolution fraction 10	Sample B1	Pearson correlation score between specific activity and relative protein abundance
49	Q1Q418	SHSP domain-containing protein	0.00E+00	0.00E+00	1.03E+04	0.72
50	A0A2C9CGR6	Thiazole synthase	0.00E+00	0.00E+00	5.62E+04	0.72
51	Q1Q679	2-oxoacid:acceptor oxidoreductase subunit alpha	0.00E+00	0.00E+00	7.35E+03	0.72
52	A0A2C9CD15	Probable glycine dehydrogenase (decarboxylating) subunit 2	0.00E+00	0.00E+00	1.82E+04	0.72
53	Q1Q7H1	50S ribomal protein L25	0.00E+00	0.00E+00	3.82E+04	0.72
54	A0A2C9CLF2	PDZ domain-containing protein	0.00E+00	0.00E+00	1.92E+04	0.72
55	Q1PXR4	Phphoenolpyruvate carboxylase	0.00E+00	0.00E+00	1.91E+04	0.72
56	Q1Q2K7	Carbamoyl-phphate synthase large chain	0.00E+00	0.00E+00	3.58E+05	0.72
57	Q1Q2K6	Probable glycine dehydrogenase (decarboxylating) subunit 1	0.00E+00	0.00E+00	2.21E+04	0.72
58	A0A6G7GWM8	Flotillin-like protein FloA	0.00E+00	0.00E+00	2.25E+04	0.72
59	Q1Q1N7	DUF3326 domain-containing protein	0.00E+00	0.00E+00	6.92E+03	0.72
60	A0A2C9CE92	Adenyluccinate lyase	0.00E+00	0.00E+00	1.63E+04	0.72
61	Q1Q4Q3	50S ribomal subunit assembly factor BipA	0.00E+00	0.00E+00	2.29E+05	0.72
62	Q1Q3W3	Arginine--tRNA ligase	0.00E+00	0.00E+00	6.65E+04	0.72
63	A0A2C9CAP5	2-dehydro-3-deoxyphphooctonate aldolase	0.00E+00	0.00E+00	5.86E+04	0.72
64	A0A6G7GXY1	Cytochrome c	0.00E+00	0.00E+00	3.35E+04	0.72
65	A0A2C9CFI7	Histidine--tRNA ligase	0.00E+00	0.00E+00	4.40E+04	0.72
66	A0A2C9CAN1	2,3-bisphphoglycerate-dependent phphoglycerate mutase	0.00E+00	0.00E+00	2.28E+04	0.72
67	Q1Q4M3	5-hydroxybenzimidazole synthase	0.00E+00	0.00E+00	8.49E+04	0.72
68	A0A2C9CI02	PilT/PilU family type 4a pilus ATPase	0.00E+00	0.00E+00	3.58E+04	0.72
69	A0A2C9CEZ2	Aspartate--tRNA(Asp/Asn) ligase	0.00E+00	0.00E+00	9.86E+04	0.72
70	A0A6G7GRR4	peptide-methionine (S)-S-oxide reductase	0.00E+00	0.00E+00	4.99E+04	0.72
71	A0A2C9CE81	Isoleucine--tRNA ligase	0.00E+00	0.00E+00	8.47E+04	0.72
72	A0A6G7GPV9	PTS sugar transporter subunit IIA	0.00E+00	0.00E+00	3.83E+04	0.72
73	Q1PZ66	DUF1858 domain-containing protein	3.00E+05	4.10E+04	6.95E+05	0.69
74	Q1Q3E2	Amidohydrolase-related domain-containing protein	0.00E+00	3.22E+04	2.01E+04	0.67
75	A0A2C9CKW5	Porphobilinogen deaminase	1.35E+05	2.12E+05	1.09E+05	0.66
76	Q1PW16	Trigger factor	2.79E+05	1.35E+05	1.66E+06	0.65
77	Q1PZN3	Chaperone protein HtpG	3.08E+04	1.12E+05	6.81E+05	0.65
78	Q1Q3V0	Phpho-2-dehydro-3-deoxyheptonate aldolase	0.00E+00	0.00E+00	5.35E+05	0.64
79	Q1Q105	3-isopropylmalate dehydratase small subunit	4.25E+04	0.00E+00	5.14E+04	0.63
80	A0A2C9CKK5	Uncharacterized protein	1.29E+04	4.27E+04	2.24E+05	0.63
81	A0A2C9CKK3	Peptide methionine sulfoxide reductase MsrA	0.00E+00	0.00E+00	1.17E+05	0.63
82	A0A2C9CFF5	PTS sugar transporter subunit IIA	0.00E+00	0.00E+00	6.31E+05	0.56
83	Q1Q4L7	TIGR04255 family protein	0.00E+00	0.00E+00	3.51E+04	0.55
84	Q1Q7P3	Class I small soluble cyt c	4.73E+06	4.07E+06	2.28E+06	0.53
85	Q1PUP9	Adenylhomocysteinase	0.00E+00	0.00E+00	1.99E+06	0.52
86	A0A2C9CFG1	Chaperonin GroEL	8.43E+04	4.04E+04	1.75E+05	0.51
87	Q1PYX1	ATP-dependent Clp protease proteolytic subunit	0.00E+00	0.00E+00	2.35E+05	0.50
88	Q1PZK4	Chaperonin GroEL	3.11E+04	0.00E+00	5.43E+04	0.50
89	A0A2C9CDZ7	S-layer protein	2.25E+05	3.36E+05	1.15E+05	0.49
90	Q1PVI8	MoxR family ATPase	0.00E+00	0.00E+00	6.55E+04	0.49
91	Q1Q2R8	3-hydroxy-5-phphonooxypentane-2,4-dione thiolase	1.39E+05	4.81E+04	8.04E+04	0.49
92	A0A2C9CN84	Radical SAM core domain-containing protein	0.00E+00	0.00E+00	1.54E+05	0.49
93	Q1PYI1	Iron-containing redox enzyme family protein	5.25E+06	4.69E+06	2.24E+06	0.48
94	Q1PZD5	Nitrite oxidoreductase subunit B	7.13E+04	4.66E+05	2.34E+06	0.47
95	A0A2C9CKI8	transketolase	6.88E+04	7.76E+04	2.67E+04	0.44
96	A0A2C9CC12	Histidinol-phphate aminotransferase	4.55E+05	1.70E+05	2.08E+05	0.43
97	Q1Q637	DNA-binding protein	4.61E+04	1.38E+05	8.70E+04	0.43
98	Q1Q7J1	Putative hydroxylamine oxidoreductase hao	1.45E+04	1.06E+05	1.77E+05	0.40
99	Q1PZD8	Nitrite oxidoreductase subunit A	3.53E+05	1.39E+06	7.02E+06	0.39
100	A0A2C9CHV5	PQQ-like beta-propeller repeat protein	8.88E+04	0.00E+00	5.05E+04	0.37
101	A0A2C9CH14	Hydrazine synthase subunit B	8.49E+07	4.62E+07	3.14E+07	0.34
102	Q1Q4Z4	Homerine dehydrogenase	1.97E+05	0.00E+00	1.03E+05	0.33
103	Q1Q2X3	Similar to beta-ketoacyl acyl carrier protein synthase II	0.00E+00	1.56E+05	1.32E+06	0.33

Protein number	Accession	Description	Relative protein abundance per fraction			Pearson correlation score
			Low-resolution fraction 9	Low-resolution fraction 10	Sample B1	between specific activity and relative protein abundance
104	Q1Q3Y3	Purine nucleide phphorylase	0.00E+00	0.00E+00	1.34E+05	0.33
105	A0A2C9CF24	Uncharacterized protein	0.00E+00	0.00E+00	1.36E+05	0.30
106	A0A2C9CD37	Peptide methionine sulfoxide reductase MsrA	7.63E+05	2.94E+04	3.17E+05	0.28
107	A0A2C9CEF3	Putative NADH dehydrogenase I chain F (1st module)	0.00E+00	0.00E+00	2.97E+05	0.27
108	Q1PUT2	PDZ domain-containing protein	0.00E+00	0.00E+00	1.21E+05	0.26
109	A0A2C9CHN2	Hydrazine synthase subunit A	1.16E+08	5.16E+07	3.49E+07	0.26
110	A0A2C9CCP8	PDZ domain-containing protein	0.00E+00	0.00E+00	1.15E+05	0.25
111	A0A2C9CDK8	Gamma-glutamyl phphate reductase	0.00E+00	0.00E+00	5.68E+04	0.25
112	Q1PZD4	Nitrite oxidoreductase subunit C	1.55E+05	5.51E+05	2.63E+06	0.24
113	A0A2C9CHM2	Hydrazine synthase subunit C	1.01E+08	4.28E+07	2.96E+07	0.24
114	A0A2C9CBX8	Glutamine synthetase	6.41E+04	2.60E+04	1.49E+04	0.24
115	Q1PV11	DUF2024 domain-containing protein	0.00E+00	0.00E+00	8.14E+04	0.23
116	Q1Q2J4	Elongation factor Ts	2.26E+05	4.03E+04	6.69E+04	0.23
117	Q1Q277	Uncharacterized protein	0.00E+00	1.68E+04	0.00E+00	0.21
118	Q1PXN5	Chemotaxis protein CheY	0.00E+00	2.17E+04	0.00E+00	0.21
119	Q1Q666	Putative transcriptional repressor	0.00E+00	1.15E+04	0.00E+00	0.21
120	A0A2C9CDI4	Glycerate 2-kinase	0.00E+00	2.07E+04	0.00E+00	0.21
121	A0A2C9CF13	Chaperonin GroEL	4.40E+04	0.00E+00	6.82E+04	0.21
122	Q1Q3W5	Glutamate synthase (NADPH) large chain	7.54E+04	1.77E+04	1.48E+04	0.16
123	Q1PVE2	Type-1 blue copper-containing cupredoxin	1.22E+05	3.76E+04	1.94E+04	0.15
124	Q1PZC8	Putative septation protein SpoVG	8.78E+04	5.86E+04	5.09E+04	0.14
125	Q1Q354	Sulfate adenylyltransferase	0.00E+00	0.00E+00	1.58E+06	0.13
126	Q1PZK3	Co-chaperonin GroES	1.21E+06	4.33E+05	6.08E+05	0.11
127	A0A2C9CHX8	Dihydroxy-acid dehydratase	0.00E+00	0.00E+00	2.22E+05	0.11
128	Q1Q4H1	Heme d1 biynthesis protein NirF	1.26E+04	8.93E+03	0.00E+00	0.10
129	Q1Q1A9	Argininuccinate synthase	3.45E+04	0.00E+00	6.95E+03	0.10
130	A0A2C9CGP5	Flavodoxin family protein	0.00E+00	0.00E+00	3.54E+04	0.09
131	A0A2C9CDL6	Uncharacterized protein	0.00E+00	0.00E+00	1.10E+06	0.07
132	A0A2C9CE26	Acetolactate synthase	3.57E+05	1.28E+05	1.88E+04	0.07
133	Q1PVQ3	Hsp20/alpha crystallin family protein	7.83E+05	2.75E+05	4.25E+04	0.07
134	Q1PW67	Peptidylprolyl isomerase	2.30E+06	4.61E+05	4.18E+05	0.06
135	A0A6G7GTJ9	Uncharacterized protein	9.51E+05	2.28E+05	2.45E+04	0.02
136	A0A2C9CKL5	Uncharacterized protein	4.77E+04	1.16E+04	0.00E+00	0.00
137	A0A2C9CHG6	CBS domain-containing protein	0.00E+00	0.00E+00	2.09E+04	0.00
138	Q1PXW3	Lon protease	0.00E+00	0.00E+00	5.23E+05	-0.01
139	Q1PY42	Co-chaperonin GroES	2.11E+06	3.01E+05	0.00E+00	-0.02
140	Q1PWY3	Chaperone protein ClpB	0.00E+00	0.00E+00	8.57E+05	-0.03
141	Q1PW64	Zinc ribbon domain protein	1.34E+05	1.18E+04	0.00E+00	-0.03
142	Q1PYI0	FMN-binding glutamate synthase family protein	7.04E+04	6.08E+03	0.00E+00	-0.03
143	Q1Q1A6	Uncharacterized protein	9.74E+05	6.01E+04	0.00E+00	-0.04
144	A0A2C9CGA0	Hsp20/alpha crystallin family protein	7.15E+05	1.01E+04	7.85E+03	-0.04
145	A0A2C9CEW4	transketolase	2.99E+05	1.08E+04	0.00E+00	-0.04
146	A0A2C9CC59	Bifunctional purine biynthesis protein PurH	2.03E+05	5.32E+03	0.00E+00	-0.05
147	A0A2C9CDL0	Beta-ketoacyl-[acyl-carrier-protein] synthase III	0.00E+00	0.00E+00	1.47E+05	-0.05
148	A0A2C9CID2	Acetylornithine aminotransferase	1.67E+05	0.00E+00	0.00E+00	-0.05
149	Q1PZI4	Nucleide diphphate kinase	7.81E+04	0.00E+00	0.00E+00	-0.05
150	A0A2C9CBY9	uroporphyrinogen-III C-methyltransferase	2.38E+05	0.00E+00	0.00E+00	-0.05
151	Q1PVG3	Cyclic nucleotide-binding domain-containing protein	2.74E+04	0.00E+00	0.00E+00	-0.05
152	A0A2C9CA80	DUF1326 domain-containing protein	2.39E+05	0.00E+00	0.00E+00	-0.05
153	A0A2C9CIP1	Flagellar hook protein FlgE	6.12E+04	0.00E+00	0.00E+00	-0.05
154	Q1PXI4	Uncharacterized protein	1.73E+05	0.00E+00	0.00E+00	-0.05
155	A0A2C9CEZ1	Transpase	2.82E+05	0.00E+00	0.00E+00	-0.05
156	Q1Q3D6	Carboxypeptidase regulatory-like domain-containing protein	2.68E+04	0.00E+00	0.00E+00	-0.05
157	A0A2C9CLC9	6-phphogluconate dehydrogenase, decarboxylating	5.95E+05	0.00E+00	0.00E+00	-0.05
158	Q1Q338	Adenylyl-sulfate reductase subunit alpha	3.92E+04	0.00E+00	0.00E+00	-0.05

Protein number	Accession	Description	Relative protein abundance per fraction			Pearson correlation score
			Low-resolution fraction 9	Low-resolution fraction 10	Sample B1	between specific activity and relative protein abundance
159	A0A2C9CDR5	Strongly similar to sigma 54 formate hydrogen lyase transcriptional activator FlhA	6.29E+04	0.00E+00	0.00E+00	-0.05
160	Q1Q416	Small heat shock protein C2	5.58E+04	0.00E+00	0.00E+00	-0.05
161	A0A2C9CK20	Valine--tRNA ligase	1.75E+05	0.00E+00	0.00E+00	-0.05
162	Q1PX47	Tryptophan synthase beta chain	1.29E+04	0.00E+00	0.00E+00	-0.05
163	A0A2C9CDU6	Uncharacterized protein	4.19E+04	0.00E+00	0.00E+00	-0.05
165	Q1Q2L2	Gluce-1-phphate thymidylyltransferase	8.78E+04	0.00E+00	0.00E+00	-0.05
166	A0A6G7GL09	site-specific DNA-methyltransferase (adenine-specific)	2.81E+05	0.00E+00	0.00E+00	-0.05
167	Q1Q7C6	Similar to ATP dependent transcriptional activator	3.95E+05	0.00E+00	0.00E+00	-0.05
168	Q1Q0T9	Hypothetical (Triheme) protein	1.26E+04	0.00E+00	0.00E+00	-0.05
169	Q1PV60	PEGA domain-containing protein	1.15E+04	0.00E+00	0.00E+00	-0.05
170	A0A2C9CHS3	3-isopropylmalate dehydratase large subunit	5.87E+04	0.00E+00	0.00E+00	-0.05
171	Q1Q0A1	Putative serine-proteinase HtrA/ DegQ/ DegS family	2.15E+04	0.00E+00	0.00E+00	-0.05
172	A0A2C9CHF8	Thioredoxin domain-containing protein	2.25E+04	0.00E+00	0.00E+00	-0.05
173	A0A2C9CAW2	MULTIHEME_CYTC domain-containing protein	2.33E+04	0.00E+00	0.00E+00	-0.05
174	Q1PZA6	Amidohydrolase	1.12E+05	0.00E+00	0.00E+00	-0.05
175	A0A2C9CCG2	Uncharacterized protein	6.25E+04	0.00E+00	0.00E+00	-0.05
176	A0A2C9CB13	Strongly similar to pyruvate:ferredoxin oxidoreductase	6.11E+04	0.00E+00	0.00E+00	-0.05
177	A0A2C9CC73	UDP-N-acetylmuramoylalanine--D-glutamate ligase	3.92E+06	0.00E+00	0.00E+00	-0.05
178	Q1Q697	Quinolinate synthase	6.19E+04	0.00E+00	0.00E+00	-0.05
179	Q1Q6A7	Cold-shock protein	4.74E+04	9.31E+04	1.48E+06	-0.06
180	Q1Q133	Transcription termination/antitermination protein NusG	1.98E+05	4.62E+04	1.59E+04	-0.08
181	Q1Q3G0	PhoU domain-containing protein	0.00E+00	0.00E+00	2.55E+05	-0.13
182	Q1Q2D8	Cysteine desulfurase	0.00E+00	0.00E+00	7.00E+04	-0.19
183	Q1Q123	Elongation factor Tu	9.03E+04	7.68E+04	6.05E+06	-0.21
184	A0A2C9CHA0	50S ribomal protein L7/L12	0.00E+00	7.62E+03	3.10E+05	-0.28
185	Q1Q1C6	PQQ-like beta-propeller repeat protein	0.00E+00	0.00E+00	7.78E+04	-0.30
186	A0A2C9CBQ3	Chaperone protein DnaK	0.00E+00	3.23E+04	4.48E+06	-0.31
187	A0A2C9CJE3	Glyco_hydro_cc domain-containing protein	0.00E+00	0.00E+00	4.78E+05	-0.33
188	Q1PYW4	Carbohydrate kinase family protein	0.00E+00	0.00E+00	5.28E+04	-0.37
189	Q1Q1N9	Putative inine-5'-monophphate dehydrogenase related protein II	0.00E+00	0.00E+00	6.31E+05	-0.37
190	Q1PY51	Scaffold protein	0.00E+00	0.00E+00	6.48E+04	-0.40
191	A0A2C9CD92	Glutamate--tRNA ligase	0.00E+00	0.00E+00	6.58E+04	-0.41
192	A0A2C9CB61	Cofactor-independent phphoglycerate mutase	0.00E+00	0.00E+00	1.34E+05	-0.41
193	Q1PUL0	Porin	0.00E+00	4.03E+04	2.09E+05	-0.42
194	Q1Q1N4	Hypothetical phphotransacetylase protein	0.00E+00	0.00E+00	6.10E+04	-0.43
195	Q1PVQ5	Putative superoxide reductase	5.67E+04	6.62E+04	4.10E+05	-0.45
196	Q1Q4X4	HlyD_D23 domain-containing protein	0.00E+00	0.00E+00	6.35E+04	-0.48
197	A0A2C9CKJ6	Exported protein	5.89E+04	6.35E+04	1.01E+05	-0.56
198	A0A2C9CBQ7	Uncharacterized protein	0.00E+00	0.00E+00	1.27E+05	-0.59
199	A0A2C9CKT8	Sulfatase-modifying factor enzyme domain-containing protein	0.00E+00	0.00E+00	1.43E+05	-0.63
200	Q1PXG4	Ferritin family protein	0.00E+00	0.00E+00	1.88E+05	-0.66
201	Q1PXG5	Thioredoxin reductase	0.00E+00	0.00E+00	3.61E+04	-0.66
202	Q1PYT0	Enolase	0.00E+00	0.00E+00	1.27E+05	-0.69
203	A0A2C9CLM3	YfdX protein	0.00E+00	0.00E+00	2.85E+05	-0.69
204	Q1Q7P0	Uncharacterized protein	8.31E+03	0.00E+00	2.70E+05	-0.72
205	Q1PXY6	phphoenolpyruvate--protein phphotransferase	0.00E+00	0.00E+00	9.09E+03	-0.72
206	A0A2C9CBT5	Riboflavin biynthesis protein RibBA	0.00E+00	0.00E+00	1.61E+04	-0.74
207	Q1Q3K7	Elongation factor P	0.00E+00	0.00E+00	3.10E+04	-0.74
208	Q1PUP8	MoaD/ThiS family protein	0.00E+00	0.00E+00	4.55E+04	-0.76
209	Q1Q5R4	Energy-dependent translational throttle protein EttA	0.00E+00	0.00E+00	4.23E+04	-0.77
210	Q1Q303	Exported protein	0.00E+00	0.00E+00	4.61E+05	-0.77
211	Q1Q7G9	30S ribomal protein S6	0.00E+00	0.00E+00	2.24E+04	-0.78
212	A0A2C9CIY0	Proline--tRNA ligase	0.00E+00	0.00E+00	4.96E+04	-0.79
213	Q1Q141	Elongation factor G	6.11E+04	0.00E+00	3.26E+05	-0.80
214	Q1Q0K4	NIF system FeS cluster assembly NifU C-terminal domain-containing protein	0.00E+00	0.00E+00	2.08E+05	-0.81

Protein number	Accession	Description	Relative protein abundance per fraction			Pearson correlation score
			Low-resolution fraction 9	Low-resolution fraction 10	Sample B1	between specific activity and relative protein abundance
215	Q1PYS0	Fructe-1,6-bisphphatase class 1	0.00E+00	0.00E+00	5.67E+04	-0.81
216	Q1PYY4	NAD-dependent formate dehydrogenase alpha subunit / selenocysteine-containing	0.00E+00	0.00E+00	1.79E+05	-0.82
217	Q1PW50	FAD-dependent oxidoreductase	0.00E+00	0.00E+00	1.31E+05	-0.82
218	A0A2C9CIU1	Glutaredoxin domain-containing protein	0.00E+00	0.00E+00	1.90E+04	-0.83
219	Q1PY41	Chaperonin GroEL	8.30E+04	0.00E+00	1.97E+05	-0.84
220	A0A2C9CHJ5	FmdE domain-containing protein	0.00E+00	0.00E+00	8.77E+04	-0.85
221	Q1PW49	NADH-quinone oxidoreductase subunit NuoF	0.00E+00	0.00E+00	3.79E+04	-0.85
222	A0A2C9CDQ2	Strongly similar to proton-translocating NADH dehydrogenase I, 51 kDa subunit (NuoF)	0.00E+00	0.00E+00	1.33E+05	-0.85
223	A0A2C9CBP2	Strongly similar to proton-translocating NADH dehydrogenase I, 51 kDa subunit (NuoF)	0.00E+00	0.00E+00	1.33E+05	-0.85
224	Q1Q0F8	Exported protein	0.00E+00	0.00E+00	1.72E+04	-0.86
225	A0A2C9CCN2	Uncharacterized protein	0.00E+00	0.00E+00	1.24E+04	-0.87
226	Q1Q6R3	Strongly similar to to proton-translocating NADH dehydrogenase I, 24 kDa subunit (NuoE)	0.00E+00	0.00E+00	1.08E+04	-0.87
265	Q1PV06	Protein tyrine phphatase family protein	7.45E+04	1.70E+04	0.00E+00	-0.97
