# Supplementary material for: Nitric oxide‐forming nitrite reductases in the anaerobic ammonium oxidizer Kuenenia stuttgartiensis
Source: FEBS Open Bio. 2025 Aug 4;15(10):1696–713. doi: 10.1002/2211-5463.70086 (PMC12485887; doi:10.1002/2211-5463.70086)
Supplement: Supplementary file 5 — Table S3. Correlation between the relative abundance of identified proteins and specific nitrite reductase activity in the fractions obtained from sample B1 that were further separated on a high‐resolution anion‐exchanger. [file FEB4-15-1696-s007.pdf]

Supplementary table 3 - Correlation between the relative abundance of identified proteins and specific nitrite reductase activity in the fractions obtained from sample B1 that were further separated on a high-resolution anion-exchanger. Pearson correlation score was calculated between the specific activity measured per fraction and the relative protein abundance measured per fraction. The data is ordered based on relative protein abundance that correlated best with the specific activity. The relative abundance of NirS correlated well with specific nitrite reductase activity. Moreover, NirS had the highest relative abundance measured in most active Fraction 9. Other proteins that showed a positive correlation are not identified as nitrite reductases. Accession numbers refer to the *K. stuttgartiensis* protein sequence database in Uniprot (entry KSMBR1).

Protein number	Accession	Description	Relative protein abundance per fraction<br>Fraction 7	Fraction 8	Fraction 9	Fraction 10	Pearson correlation score<br>between specific activity and relative protein abundance
**1**	**Q1Q4F5**	**Strongly similar to cd1 nitrite reductase NirS**	**2.76E+06**	**2.90E+07**	**4.38E+07**	**3.01E+07**	**0.87**
2	A0A2C9CAZ0	Uncharacterized protein	0.00E+00	1.99E+03	1.46E+04	0.00E+00	0.77
3	Q1Q3W5	Glutamate synthase (NADPH) large chain	0.00E+00	7.97E+04	9.63E+05	7.21E+04	0.74
4	Q1PYI7	Protein RecA	0.00E+00	0.00E+00	5.76E+03	0.00E+00	0.69
5	A0A2C9CN84	Radical SAM core domain-containing protein	0.00E+00	0.00E+00	1.13E+04	0.00E+00	0.69
6	Q1PYI1	Iron-containing redox enzyme family protein	0.00E+00	0.00E+00	1.33E+05	0.00E+00	0.69
7	Q1Q2D9	Putative periplasmic serine endoprotease DegP-like	0.00E+00	0.00E+00	1.23E+05	0.00E+00	0.69
8	Q1PXC8	Peptidylprolyl isomerase	0.00E+00	0.00E+00	6.99E+05	1.22E+04	0.69
9	Q1PW15	ATP-dependent Clp protease proteolytic subunit	4.77E+04	4.29E+04	1.24E+05	0.00E+00	0.64
10	Q1PX48	Hydroxylamine oxidoreductase	3.46E+06	8.54E+06	4.67E+06	3.23E+06	0.60
11	Q1Q6A7	Cold-shock protein	1.52E+05	1.29E+05	2.74E+05	0.00E+00	0.55
12	Q1PZC8	Putative septation protein SpoVG	9.76E+04	1.01E+05	1.37E+05	2.03E+04	0.55
13	Q1Q338	Adenylyl-sulfate reductase subunit alpha	0.00E+00	3.74E+04	0.00E+00	0.00E+00	0.40
14	Q1Q201	Integration ht factor subunit beta	1.02E+05	9.95E+04	2.73E+05	2.90E+05	0.27
15	Q1PZD5	Nitrite oxidoreductase subunit B	5.08E+06	8.28E+06	3.18E+06	8.15E+05	0.22
16	Q1Q123	Elongation factor Tu	4.66E+06	4.65E+06	3.99E+06	2.06E+06	0.16
17	Q1PZD8	Nitrite oxidoreductase subunit A	2.00E+07	2.97E+07	1.09E+07	3.63E+06	0.13
18	A0A2C9CF13	Chaperonin GroEL	5.76E+04	5.84E+04	3.61E+04	0.00E+00	0.08
19	A0A2C9CBQ3	Chaperone protein DnaK	2.70E+05	2.03E+05	1.92E+05	0.00E+00	0.03
20	Q1Q7P0	Uncharacterized protein	1.20E+05	8.92E+04	8.57E+04	0.00E+00	0.03
21	Q1PZD4	Nitrite oxidoreductase subunit C	8.14E+06	9.33E+06	3.96E+06	4.99E+05	0.01
22	A0A2C9CFG1	Chaperonin GroEL	6.26E+04	5.84E+04	3.61E+04	0.00E+00	0.01
23	Q1PY41	Chaperonin GroEL	1.21E+05	9.60E+04	7.75E+04	0.00E+00	0.00
24	Q1Q0K4	NIF system FeS cluster assembly NifU C-terminal domain-containing protein	2.81E+04	0.00E+00	2.86E+04	0.00E+00	-0.05
25	A0A2C9CHN2	Hydrazine synthase subunit A	1.46E+06	1.43E+06	7.35E+05	1.79E+05	-0.08
26	Q1PVQ5	Putative superoxide reductase	6.47E+04	4.25E+04	4.04E+04	0.00E+00	-0.09
27	A0A2C9CH14	Hydrazine synthase subunit B	1.69E+06	1.54E+06	7.27E+05	1.64E+05	-0.16
28	Q1Q3G0	PhoU domain-containing protein	3.58E+04	4.70E+04	0.00E+00	0.00E+00	-0.17
29	A0A2C9CHM2	Hydrazine synthase subunit C	2.51E+06	2.06E+06	9.71E+05	2.05E+05	-0.23
30	A0A2C9CKJ6	Exported protein	6.26E+04	3.91E+04	2.31E+04	0.00E+00	-0.31
31	Q1PXW3	Lon protease	2.74E+05	2.22E+05	2.74E+04	0.00E+00	-0.37
32	Q1PYX1	ATP-dependent Clp protease proteolytic subunit	1.78E+05	1.10E+05	0.00E+00	0.00E+00	-0.53
33	A0A2C9CLM3	YfdX protein	1.24E+07	7.39E+06	3.19E+05	1.16E+04	-0.53
34	A0A2C9CDL6	Uncharacterized protein	2.22E+06	7.55E+05	4.48E+05	0.00E+00	-0.57
35	Q1PZD7	Uncharacterized protein	8.53E+04	2.54E+04	0.00E+00	0.00E+00	-0.69
36	A0A2C9CHG6	CBS domain-containing protein	4.53E+04	0.00E+00	0.00E+00	0.00E+00	-0.77
37	Q1PYT0	Enolase	4.59E+04	0.00E+00	0.00E+00	0.00E+00	-0.77
38	A0A2C9CDQ2	Strongly similar to proton-translocating NADH dehydrogenase I, (NuoF)	1.17E+04	0.00E+00	0.00E+00	0.00E+00	-0.77
39	A0A2C9CBP2	Strongly similar to proton-translocating NADH dehydrogenase I, (NuoF)	1.17E+04	0.00E+00	0.00E+00	0.00E+00	-0.77
40	A0A2C9CCN2	Uncharacterized protein	7.63E+04	0.00E+00	0.00E+00	0.00E+00	-0.77
