# Supplementary material for: Nitric oxide‐forming nitrite reductases in the anaerobic ammonium oxidizer Kuenenia stuttgartiensis
Source: FEBS Open Bio. 2025 Aug 4;15(10):1696–713. doi: 10.1002/2211-5463.70086 (PMC12485887; doi:10.1002/2211-5463.70086)
Supplement: Supplementary file 6 — Table S4. Correlation between the relative abundance of identified proteins and specific nitrite reductase activity in three samples obtained with low‐resolution column chromatography: FT, sample A, and low‐resolution fraction 6. [file FEB4-15-1696-s006.pdf]

Supplementary table 4 - Correlation between the relative abundance of identified proteins and specific nitrite reductase activity in three samples obtained with low-resolution column chromatography: FT, sample A, and low-resolution fraction 6. Pearson correlation score was calculated between the specific activity measured per fraction and the relative protein abundance measured per fraction. The data is ordered based on relative protein abundance that correlated best with the specific activity. None of the proteins that showed a positive correlation are identified as nitrite reductases. Accession numbers refer to the *K. stuttgartiensis* protein sequence database in Uniprot (entry KSMBR1).

Protein number	Accession	Description	Relative protein abundance per fraction FT	sample A	Low-resolution fraction 6	Pearson correlation score between specific activity and relative protein abundance
1	Q1PYG3	Adenylyl-sulfate kinase	0.00E+00	3.95E+01	2.77E+01	1.00
2	Q1PXM5	Chemotaxis protein CheY-like protein	0.00E+00	5.53E+03	8.16E+01	1.00
3	A0A2C9CD35	Methylthioribose-1-phosphate isomerase	0.00E+00	5.24E+02	1.58E+02	1.00
4	Q1Q1E9	Protein-arginine kinase	0.00E+00	1.36E+02	9.98E+01	1.00
5	Q1Q243	Putative deoxyribonuclease YcfH / radical SAM domain protein	0.00E+00	3.68E+02	7.53E+01	1.00
6	Q1PUP8	Putative molybdopterin synthase subunit 1	9.57E+00	0.00E+00	1.27E+01	1.00
7	Q1Q0C4	2-amino-4-hydroxy-6-hydroxymethyldihydropteridine diphosphokinase	0.00E+00	3.48E+02	3.03E+02	1.00
8	Q1Q7G3	3-isopropylmalate dehydrogenase	0.00E+00	1.22E+02	6.38E+01	1.00
9	Q1Q7G2	AMMECR1 domain-containing protein	0.00E+00	5.21E+01	2.50E+01	1.00
10	Q1PYX1	ATP-dependent Clp protease proteolytic subunit	0.00E+00	2.46E+02	1.85E+02	1.00
11	Q1Q504	Branched-chain-amino-acid aminotransferase	0.00E+00	5.00E+01	1.76E+01	1.00
12	Q1PYY7	Carbon monoxide dehydrogenase accessory protein CooC	0.00E+00	5.34E+01	1.90E+01	1.00
13	Q1PZB8	Chemotaxis protein CheY	0.00E+00	3.86E+02	2.46E+02	1.00
14	Q1Q516	Diheme cytochrome c	0.00E+00	3.73E+03	2.70E+03	1.00
15	Q1Q0K3	DUF1858 domain-containing protein	0.00E+00	1.09E+02	5.22E+01	1.00
16	Q1Q7J5	DUF1858 domain-containing protein	0.00E+00	2.55E+03	5.74E+02	1.00
17	Q1Q6R5	Flagellar protein FliL	0.00E+00	2.39E+02	9.07E+01	1.00
18	Q1Q106	Flavodoxin-like domain-containing protein	0.00E+00	3.31E+01	3.01E+01	1.00
19	Q1PZ82	Glyceraldehyde-3-phosphate dehydrogenase	1.73E+01	0.00E+00	2.72E+01	1.00
20	Q1PXF9	Glycogen branching enzyme, GH-57-type, archaeal	0.00E+00	2.25E+01	1.85E+01	1.00
21	Q1Q012	HEAT repeat domain-containing protein	0.00E+00	9.48E+01	7.33E+01	1.00
22	Q1Q5K4	histidine kinase	0.00E+00	5.33E+01	5.17E+01	1.00
23	Q1PWX7	Imidazole glycerol phosphate synthase subunit HisH	0.00E+00	1.74E+02	7.74E+01	1.00
24	Q1PXU6	Insulinase family protein	0.00E+00	6.32E+02	5.60E+02	1.00
25	Q1Q201	Integration host factor subunit beta	0.00E+00	8.33E+01	7.87E+01	1.00
26	A0A2C9CC85	Large ribosomal subunit protein bL19	0.00E+00	7.47E+01	4.06E+01	1.00
27	Q1PVS7	Metallo-beta-lactamase family protein, RNA-specific	0.00E+00	3.90E+01	2.78E+01	1.00
28	Q1Q1J9	Methylthioribose-1-phosphate isomerase	0.00E+00	5.32E+02	3.89E+02	1.00
29	Q1PX75	Methyltransferase	0.00E+00	1.04E+03	1.51E+02	1.00
30	Q1Q2I9	MOSC domain-containing protein	9.00E+01	3.21E+02	0.00E+00	1.00
31	Q1Q242	N-acetylglutamate synthase	0.00E+00	6.99E+01	4.69E+01	1.00
32	Q1Q0K4	NIF system FeS cluster assembly NifU C-terminal domain-containing protein	0.00E+00	7.83E+01	4.64E+01	1.00
33	Q1Q7F3	peptidoglycan lytic exotransglycosylase	0.00E+00	2.73E+02	2.38E+02	1.00
34	Q1PY30	Phosphoribosylamine--glycine ligase	0.00E+00	1.99E+02	7.48E+01	1.00
35	Q1PYD1	PilZ domain-containing protein	0.00E+00	1.10E+02	7.75E+01	1.00
36	Q1Q0Q0	Putative acriflavine resistance protein F	0.00E+00	6.01E+03	5.26E+02	1.00
37	Q1Q7J1	Putative hydroxylamine oxidoreductase hao	0.00E+00	4.46E+01	4.17E+01	1.00
38	Q1PXM2	Putative N-acetyl-alpha-D-glucosaminyl L-malate deacetylase 1	0.00E+00	3.39E+02	6.88E+01	1.00
39	Q1Q7A2	Putative serine/threonine specific protein phosphatase 2	0.00E+00	2.35E+02	1.03E+02	1.00
40	Q1PZS9	Pyruvoyl-dependent arginine decarboxylase AaxB	0.00E+00	1.76E+02	1.75E+02	1.00
41	Q1PZ79	Ribulose-phosphate 3-epimerase	0.00E+00	1.78E+02	1.70E+02	1.00
42	Q1PVM6	Strongly similar to peroxiredoxin (Thioredoxin peroxidase)	0.00E+00	4.43E+02	1.87E+02	1.00
43	Q1Q4I1	Type II toxin-antitoxin system RelE/ParE family toxin	0.00E+00	2.02E+02	1.48E+02	1.00
44	Q1PZV2	Tyrosine--tRNA ligase	0.00E+00	4.85E+01	1.35E+01	1.00
45	A0A2C9CCM4	UDP-N-acetylglucosamine 1-carboxyvinyltransferase	0.00E+00	6.36E+01	4.11E+01	1.00
46	A0A2C9CGT6	Uncharacterized protein	0.00E+00	7.85E+01	4.85E+01	1.00
47	Q1PW80	Uncharacterized protein	0.00E+00	1.91E+02	7.38E+01	1.00
48	Q1Q6L5	Uncharacterized protein	0.00E+00	7.10E+01	5.69E+01	1.00
49	Q1PV39	Secondary thiamine-phosphate synthase enzyme	0.00E+00	5.55E+02	8.76E+01	1.00

Protein number	Accession	Description	Relative protein abundance per fraction FT	sample A	Low-resolution fraction 6	Pearson correlation score between specific activity and relative protein abundance
50	A0A2C9CAF9	2-C-methyl-D-erythritol 4-phosphate cytidylyltransferase	0.00E+00	9.38E+01	3.11E+01	1.00
51	Q1PZP7	Uncharacterized protein	0.00E+00	3.40E+02	9.31E+01	1.00
52	Q1PWY2	4-hydroxy-tetrahydrodipicolinate reductase	4.05E+01	1.76E+02	6.27E+01	1.00
53	Q1PY54	Methyltransferase domain-containing protein	1.32E+02	5.55E+02	2.21E+02	1.00
54	Q1PXH9	ADP-dependent (S)-NAD(P)H-hydrate dehydratase	2.56E+01	7.64E+01	2.97E+01	1.00
55	Q1PZK4	Chaperonin GroEL	3.79E+01	1.80E+03	1.50E+02	1.00
56	Q1PX08	Regulatory protein P-II for glutamine synthetase	3.02E+01	4.75E+01	3.05E+01	0.99
57	Q1PZD9	Blue copper protein	2.48E+01	3.76E+02	1.75E+01	0.99
58	Q1Q0Y3	Glucose-6-phosphate isomerase	3.71E+01	6.01E+01	3.60E+01	0.98
59	Q1Q1E8	Chaperone protein ClpB	5.15E+01	2.33E+02	1.20E+02	0.97
60	Q1PV08	Hemerythrin-like domain-containing protein	3.54E+01	5.10E+02	2.31E+02	0.96
61	Q1PX62	DUF4438 domain-containing protein	4.89E+01	6.77E+01	5.73E+01	0.95
62	Q1PZC1	Addiction module component	7.47E+01	3.17E+02	2.15E+01	0.95
63	Q1Q2L9	Aspartate-semialdehyde dehydrogenase	2.96E+01	6.91E+01	1.70E+01	0.93
64	Q1Q2J0	Molybdenum cofactor biosynthesis protein B	3.40E+02	3.75E+02	3.58E+02	0.92
65	Q1PXR8	Serine-pyruvate aminotransferase	7.08E+02	1.45E+03	4.31E+02	0.91
66	Q1Q3C3	Putative NADH dehydrogenase/NAD(P)H nitroreductase	2.38E+02	5.66E+02	1.08E+02	0.91
67	Q1Q648	Bifunctional protein FolD	2.69E+02	6.05E+02	1.35E+02	0.91
68	Q1Q087	Pyrroline-5-carboxylate reductase	2.75E+01	1.75E+02	1.14E+02	0.89
69	Q1Q646	Probable cytosol aminopeptidase	3.94E+01	4.70E+01	4.42E+01	0.87
70	Q1Q131	Large ribosomal subunit protein uL1	2.07E+02	3.94E+02	9.42E+01	0.86
71	Q1Q6K3	Strongly similar to exoribonuclease YhaM	7.52E+01	1.10E+02	5.36E+01	0.86
72	Q1PVK4	Similar to phosphoglucomutase	3.06E+01	8.38E+01	6.90E+01	0.81
73	Q1Q2J4	Elongation factor Ts	8.74E+02	2.74E+03	2.27E+03	0.80
74	Q1Q156	Adenylate kinase	1.79E+01	1.65E+03	1.29E+03	0.78
75	A0A2C9CKJ6	Exported protein	1.72E+02	3.43E+03	2.72E+03	0.77
76	Q1Q353	DNA polymerase beta	8.24E+01	1.47E+02	1.36E+02	0.74
77	Q1PXP1	Acetoacetate metabolism regulatory protein AtoC	1.64E+01	4.12E+01	4.29E+01	0.58
78	Q1PV34	2',3'-cyclic-nucleotide 2'-phosphodiesterase	8.20E+01	5.74E+02	6.16E+02	0.57
79	Q1PYR9	Putative fructose-biphosphate aldolase	2.81E+02	3.04E+02	1.13E+02	0.46
80	Q1PXW4	N-acetyl-gamma-glutamyl-phosphate reductase	1.55E+02	1.69E+02	4.37E+01	0.46
81	Q1Q129	Large ribosomal subunit protein bL12	4.21E+01	2.07E+02	2.93E+02	0.33
82	Q1PVQ5	Putative superoxide reductase	3.05E+01	9.18E+01	1.29E+02	0.29
83	Q1Q0V3	Aconitate hydratase	2.56E+02	2.29E+02	9.54E+01	0.21
**84**	**Q1Q4F5**	**Nitrite reductase; strongly similar to NirS**	**3.62E+01**	**7.17E+01**	**1.01E+02**	**0.20**
85	Q1Q3U7	LL-diaminopimelate aminotransferase	7.87E+02	5.93E+02	5.99E+01	0.11
86	Q1Q652	NAD-dependent epimerase/dehydratase domain-containing protein	1.52E+02	1.19E+02	4.06E+01	0.07
87	Q1PVQ3	Putative small heat shock protein	1.19E+02	4.42E+02	9.17E+02	0.04
88	Q1Q0L1	Cobalt-precorrin-4 C(11)-methyltransferase	3.19E+01	3.80E+01	4.98E+01	-0.03
89	Q1PYI8	Alanine--tRNA ligase	6.63E+01	1.35E+02	3.78E+02	-0.16
90	Q1PY42	Co-chaperonin GroES	1.48E+02	1.21E+03	9.27E+03	-0.26
91	Q1PY25	Thioredoxin	1.71E+02	1.08E+02	6.74E+01	-0.27
92	Q1PW67	Peptidylprolyl isomerase	1.42E+01	1.06E+02	9.48E+02	-0.28
93	Q1Q5R5	Aspartokinase	2.80E+02	3.07E+02	7.27E+02	-0.31
94	Q1PUK7	Formate--tetrahydrofolate ligase	1.68E+03	9.97E+02	6.47E+02	-0.33
95	Q1PYA3	Putative beta-lactamase-inhibitor-like PepSY-like domain-containing protein	6.17E+02	2.04E+02	6.47E+01	-0.42
96	Q1Q3B4	HIT domain-containing protein	5.69E+01	2.13E+01	1.05E+01	-0.44
97	Q1Q637	Exported protein	1.84E+02	6.90E+01	3.50E+01	-0.44
98	Q1Q0T4	Hydrazine synthase subunit C	6.52E+02	2.11E+02	4.70E+03	-0.44
99	Q1Q302	UDP-glucose 6-dehydrogenase	4.48E+01	2.04E+01	1.36E+01	-0.45
100	Q1PW28	Uncharacterized protein	7.56E+03	1.82E+03	3.93E+02	-0.47
101	Q1Q177	Pyruvate, phosphate dikinase	9.35E+01	7.29E+01	2.26E+02	-0.48
102	Q1Q1N5	Acetate-CoA ligase [ADP-forming] I	3.41E+02	7.18E+01	2.53E+01	-0.51
103	Q1PZB4	CSD domain-containing protein	2.31E+02	9.55E+01	7.46E+01	-0.53
104	Q1PUT0	glucose-1-phosphate adenylyltransferase	9.80E+01	3.47E+01	2.54E+01	-0.53

Protein number	Accession	Description	Relative protein abundance per fraction FT	sample A	Low-resolution fraction 6	Pearson correlation score between specific activity and relative protein abundance
105	Q1Q3W5	Glutamate synthase (NADPH) large chain	5.82E+02	1.52E+02	9.52E+01	-0.54
106	A0A2C9CJF3	DUF1318 domain-containing protein	7.59E+02	1.21E+02	6.04E+01	-0.56
107	Q1PZE4	Blue copper protein	3.79E+03	4.08E+02	1.15E+02	-0.57
108	Q1PXI6	Polymerase nucleotidyl transferase domain-containing protein	2.08E+02	2.96E+01	1.56E+01	-0.57
109	A0A2C9CKP3	Cobalt-precorrin-3b C17-methyltransferase	6.69E+01	3.69E+01	3.53E+01	-0.59
110	Q1Q2J2	Ribosome-recycling factor	1.40E+03	1.13E+02	5.22E+01	-0.59
111	Q1Q117	Phosphoribosylaminoimidazole-succinocarboxamide synthase	1.03E+03	9.61E+01	5.65E+01	-0.60
112	Q1Q2R9	Putative sorbitol dehydrogenase	1.53E+03	3.30E+01	2.63E+01	-0.62
113	Q1PUK4	S-adenosylmethionine synthase	7.13E+03	3.23E+01	2.63E+01	-0.63
114	Q1Q0T9	Hypothetical (Triheme) protein	2.00E+04	6.86E+01	8.77E+01	-0.63
115	A0A2C9CE24	Serine hydroxymethyltransferase	2.75E+03	2.07E+01	2.52E+01	-0.63
116	Q1Q7I1	Outer membrane protein (OmpH-like)	3.41E+03	6.89E+01	7.91E+01	-0.63
117	Q1Q5X6	Peptidyl-prolyl cis-trans isomerase ppiD	6.78E+03	1.06E+02	1.38E+02	-0.63
118	Q1PZC8	Putative septation protein SpoVG	3.99E+02	8.34E+01	8.88E+01	-0.64
119	Q1PYU2	GTPase	6.07E+02	1.61E+01	2.87E+01	-0.64
120	Q1PY19	tRNA-splicing ligase RtcB	3.40E+02	5.84E+01	6.62E+01	-0.64
121	Q1PUP1	Asl1-like glycosyl hydrolase catalytic domain-containing protein	7.76E+02	4.34E+02	4.81E+02	-0.72
122	Q1PY86	PepSY domain-containing protein	1.06E+03	6.47E+01	2.43E+02	-0.75
123	Q1Q0Y1	Cytochrome c551 peroxidase	5.77E+01	1.69E+01	2.48E+01	-0.76
124	Q1Q3B7	Exported protein	2.09E+03	1.81E+02	6.39E+02	-0.79
125	Q1Q467	LamG-like jellyroll fold domain-containing protein	2.27E+02	1.41E+01	7.00E+01	-0.80
126	Q1Q664	Dihydrolipoamide acetyltransferase component of pyruvate dehydrogenase complex	1.54E+02	9.30E+01	1.12E+02	-0.83
127	Q1PZE3	Carboxypeptidase regulatory-like domain-containing protein	2.99E+02	1.72E+02	2.16E+02	-0.86
128	Q1Q4R9	NYN domain-containing protein	6.17E+01	2.61E+01	3.89E+01	-0.86
129	Q1Q4Z1	Cytochrome c	5.60E+02	2.78E+02	6.87E+02	-0.90
130	Q1PZD5	Nitrite oxidoreductase subunit B	4.71E+01	3.24E+01	4.57E+01	-1.00
131	Q1PZB1	Aminomethyltransferase	0.00E+00	1.93E+01	1.71E+02	-1.00
132	Q1Q3S4	Cobalamin biosynthesis precorrin-8X methylmutase CobH/CbiC domain-containing protein	0.00E+00	2.57E+02	2.65E+02	-1.00
133	Q1Q449	hydroxymethylpyrimidine kinase	1.49E+02	0.00E+00	8.94E+00	-1.00
134	Q1PVN8	Putative molybdopterin oxidoreductase, molybdopterin-containing subunit	1.83E+02	0.00E+00	2.12E+01	-1.00
135	Q1Q3U6	Tryptophan--tRNA ligase	0.00E+00	2.42E+02	1.52E+03	-1.00
136	Q1Q6D5	Uncharacterized protein	0.00E+00	3.55E+01	1.07E+02	-1.00
137	Q1Q4R8	Bifunctional chorismate mutase/prephenate dehydratase	0.00E+00	6.22E+01	1.75E+02	-1.00
138	Q1PZ66	DUF1858 domain-containing protein	0.00E+00	1.29E+02	1.47E+03	-1.00
139	Q1PVV7	Similar to glutamate-1-semialdehyde 2,1-aminomutase	0.00E+00	1.18E+02	5.44E+02	-1.00
140	Q1Q2R8	3-hydroxy-5-phosphonooxypentane-2,4-dione thiolase	0.00E+00	1.05E+02	5.90E+02	-1.00
141	Q1Q122	3-isopropylmalate dehydratase large subunit	0.00E+00	2.17E+01	2.99E+01	-1.00
142	Q1Q7P0	4Fe-4S ferredoxin	0.00E+00	1.48E+02	2.20E+02	-1.00
143	Q1Q1D3	6-carboxy-5,6,7,8-tetrahydropterin synthase	0.00E+00	7.72E+01	1.29E+02	-1.00
144	Q1Q2A1	ABC transporter substrate-binding protein	0.00E+00	1.09E+02	5.09E+02	-1.00
145	Q1PZV6	Adenine phosphoribosyltransferase	0.00E+00	2.06E+02	2.91E+02	-1.00
146	Q1Q338	Adenylylsulfate reductase alpha-subunit	0.00E+00	3.02E+02	1.03E+03	-1.00
147	Q1Q6F6	Alanine racemase	0.00E+00	1.18E+01	9.71E+01	-1.00
148	Q1Q1A1	Aldehyde oxidase/xanthine dehydrogenase	0.00E+00	4.15E+01	5.68E+01	-1.00
149	Q1PZS8	Arginine biosynthesis bifunctional protein ArgJ	0.00E+00	3.35E+03	3.54E+03	-1.00
150	Q1PV06	Beta-lactamase hydrolase-like protein	0.00E+00	1.41E+01	7.03E+02	-1.00
151	Q1Q3D6	Carboxypeptidase regulatory-like domain-containing protein	0.00E+00	2.38E+02	7.86E+02	-1.00
152	Q1Q1B0	CBS domain-containing protein	6.36E+01	3.89E+01	0.00E+00	-1.00
153	Q1PXW0	Chaperonin GroEL	0.00E+00	3.59E+01	1.20E+02	-1.00
154	Q1PZY2	Chromosome partition protein Smc	5.09E+01	0.00E+00	4.40E+01	-1.00
155	Q1Q5P8	citramalate synthase	0.00E+00	4.85E+01	9.91E+01	-1.00
156	Q1Q7P3	Class I small soluble cyt c	0.00E+00	7.09E+01	1.65E+03	-1.00
157	Q1PZK3	Co-chaperonin GroES	0.00E+00	2.26E+02	8.10E+02	-1.00
158	Q1Q2R6	Cysteine synthase A, O-acetylserine sulfhydrolase A subunit	0.00E+00	6.51E+01	8.47E+02	-1.00
159	Q1PZY5	Cytochrome c551 peroxidase	1.52E+02	1.04E+02	0.00E+00	-1.00

Protein number	Accession	Description	Relative protein abundance per fraction FT	sample A	Low-resolution fraction 6	Pearson correlation score between specific activity and relative protein abundance
160	Q1Q787	Cytochrome p450 hydroxylase MmcK-like protein	0.00E+00	5.11E+01	7.61E+01	-1.00
161	Q1PZY1	D-3-phosphoglycerate dehydrogenase	0.00E+00	9.77E+00	1.99E+02	-1.00
162	Q1Q5N0	Diaminopimelate decarboxylase	0.00E+00	2.87E+02	4.11E+02	-1.00
163	Q1PX65	DNA ligase	0.00E+00	2.05E+01	1.25E+02	-1.00
164	Q1PYZ6	DNA topoisomerase 1	0.00E+00	8.38E+00	2.46E+01	-1.00
165	Q1PUL9	DUF1015 domain-containing protein	0.00E+00	4.84E+01	5.76E+01	-1.00
166	Q1Q7G7	DUF1611 domain-containing protein	0.00E+00	1.15E+02	4.11E+02	-1.00
167	Q1PV11	DUF2024 domain-containing protein	3.96E+01	0.00E+00	1.49E+01	-1.00
168	Q1Q7J2	DUF2249 domain-containing protein	0.00E+00	4.25E+02	7.16E+02	-1.00
169	Q1Q598	DUF262 domain-containing protein	0.00E+00	4.09E+02	5.38E+02	-1.00
170	A0A2C9CI86	DUF3024 domain-containing protein	0.00E+00	2.10E+02	3.32E+02	-1.00
171	Q1Q1R5	DUF4388 domain-containing protein	0.00E+00	8.04E+01	9.38E+01	-1.00
172	Q1Q141	Elongation factor G	0.00E+00	1.81E+02	1.85E+02	-1.00
173	A0A2C9CCN1	Fragment of pimeloyl-CoA synthetase (Part 2)	2.98E+01	0.00E+00	1.08E+01	-1.00
174	Q1PYG4	GDP-L-fucose synthase	0.00E+00	1.33E+02	1.52E+02	-1.00
175	Q1PX01	GMP synthase [glutamine-hydrolyzing]	0.00E+00	1.33E+02	3.25E+02	-1.00
176	Q1Q060	GTPase Obg	0.00E+00	6.26E+01	6.46E+01	-1.00
177	Q1PY26	Indole-3-glycerol phosphate synthase	0.00E+00	5.84E+01	3.11E+02	-1.00
178	Q1PZD0	Ion-translocating oxidoreductase complex subunit B	0.00E+00	9.70E+01	4.16E+02	-1.00
179	Q1PYH4	Malonyl CoA-acyl carrier protein transacylase	0.00E+00	1.38E+01	1.02E+02	-1.00
180	Q1Q6F1	Methyltransferase domain-containing protein	0.00E+00	1.37E+02	2.38E+02	-1.00
181	Q1Q604	N-acetylmuramic acid 6-phosphate etherase	5.40E+03	3.65E+01	0.00E+00	-1.00
182	Q1Q4I3	NAD(P)H-hydrate epimerase	0.00E+00	3.30E+01	3.73E+02	-1.00
183	A0A2C9CMW4	NAD-reducing hydrogenase HoxS subunit beta	0.00E+00	4.22E+01	5.57E+01	-1.00
184	Q1PZI4	Nucleoside diphosphate kinase	0.00E+00	1.71E+02	7.44E+02	-1.00
185	Q1Q1G1	Orotidine 5'-phosphate decarboxylase	3.98E+02	2.82E+01	0.00E+00	-1.00
186	Q1PV60	PEGA domain-containing protein	0.00E+00	2.80E+02	5.37E+02	-1.00
187	Q1PY73	pEK499-p136 HEPN domain-containing protein	0.00E+00	3.63E+01	7.15E+02	-1.00
188	Q1PYD2	Peptidyl-prolyl cis-trans isomerase	4.62E+02	2.63E+00	0.00E+00	-1.00
189	Q1Q6P8	Phosphoesterase	0.00E+00	8.12E+01	1.71E+02	-1.00
190	Q1Q013	phosphomannomutase	4.44E+02	5.16E+01	0.00E+00	-1.00
191	Q1Q3G2	Phosphoribosylglycinamide formyltransferase	1.05E+03	0.00E+00	1.58E+02	-1.00
192	Q1Q6R0	Prephenate dehydrogenase	0.00E+00	5.25E+02	1.33E+03	-1.00
193	Q1Q355	Putative adenylylsulfate reductase chain B	0.00E+00	4.75E+02	7.33E+02	-1.00
194	Q1PVG3	Putative catabolite gene activator (CAMP receptor protein)	0.00E+00	8.10E+01	1.88E+02	-1.00
195	Q1Q2D9	Putative periplasmic serine endoprotease DegP-like	0.00E+00	6.05E+01	9.45E+01	-1.00
196	Q1PVM4	Putative UDP-N-acetylglucosamine pyrophosphorylase	0.00E+00	9.43E+00	1.93E+02	-1.00
197	Q1Q552	Pyruvate:ferredoxin (Flavodoxin) oxidoreductase	0.00E+00	1.09E+02	1.80E+02	-1.00
198	Q1Q045	Radical SAM domain heme biosynthesis protein	0.00E+00	3.41E+01	8.49E+01	-1.00
199	Q1PZE1	Serine hydroxymethyltransferase	1.30E+03	2.74E+02	0.00E+00	-1.00
200	Q1PXI1	siroheme decarboxylase	0.00E+00	1.81E+01	5.61E+01	-1.00
201	Q1Q728	SpoVT-AbrB domain-containing protein	0.00E+00	1.55E+02	1.77E+02	-1.00
202	Q1Q0W8	Strongly similar to small heat shock protein	0.00E+00	7.45E+02	1.29E+03	-1.00
203	Q1Q0I7	Sulfur carrier protein ThiS adenylyltransferase	0.00E+00	1.83E+01	2.84E+01	-1.00
204	Q1Q1K9	Threonine synthase	1.89E+03	6.65E+01	0.00E+00	-1.00
205	Q1PY20	Thymidylate kinase	0.00E+00	4.59E+01	1.28E+02	-1.00
206	Q1PX64	TldD protein, part of proposed TldE/TldD proteolytic complex	0.00E+00	1.44E+01	3.89E+02	-1.00
207	Q1Q775	Transaldolase	0.00E+00	1.68E+02	1.47E+03	-1.00
208	A0A6G7GTJ9	Transcription factor CBF/NF-Y/archaeal histone domain-containing protein	0.00E+00	5.20E+02	2.52E+03	-1.00
209	Q1Q088	transketolase	0.00E+00	3.03E+01	3.07E+02	-1.00
210	Q1PX46	Tryptophan synthase alpha chain	0.00E+00	1.86E+02	1.93E+03	-1.00
211	Q1PX47	Tryptophan synthase beta chain	0.00E+00	4.26E+02	9.24E+02	-1.00
212	Q1Q6I9	Uncharacterized protein	0.00E+00	2.10E+02	6.85E+02	-1.00
213	Q1PXU5	Uncharacterized protein	0.00E+00	2.25E+02	2.88E+02	-1.00
214	Q1Q2E9	Conserved hypothetical iron sulfur protein	0.00E+00	8.39E+01	3.29E+02	-1.00

Protein number	Accession	Description	Relative protein abundance per fraction			Pearson correlation score
			FT	sample A	Low-resolution fraction 6	between specific activity and relative protein abundance
215	Q1PXU8	Conserved hypothetical thioredoxin protein	1.00E+02	0.00E+00	3.64E+01	-1.00
216	Q1Q7J4	Cupin type-2 domain-containing protein	0.00E+00	1.26E+02	2.25E+03	-1.00
217	Q1Q5M8	Dissimilatory sulfite reductase (Desulfoviridin), alpha and beta subunits	1.79E+04	0.00E+00	8.43E+01	-1.00
218	Q1PX19	DJ-1/PfpI domain-containing protein	5.07E+02	0.00E+00	7.33E+00	-1.00
219	Q1PUZ2	Glucose-6-phosphate 1-dehydrogenase	2.12E+01	0.00E+00	9.54E+00	-1.00
220	Q1Q0T2	Hydrazine synthase subunit A	0.00E+00	5.14E+01	7.65E+03	-1.00
221	Q1PW61	Imidazole glycerol phosphate synthase subunit HisF	0.00E+00	2.96E+02	4.15E+02	-1.00
222	Q1PW68	Ketol-acid reductoisomerase (NADP(+))	1.30E+04	4.50E+00	0.00E+00	-1.00
223	Q1Q1W6	LamG-like jellyroll fold domain-containing protein	0.00E+00	7.73E+01	3.04E+02	-1.00
224	Q1Q7J3	Mannose-6-phosphate isomerase	0.00E+00	2.10E+01	2.66E+02	-1.00
225	Q1PZD8	Nitrite oxidoreductase subunit A	1.03E+02	0.00E+00	4.21E+01	-1.00
226	Q1PVV8	Similar to methionyl-tRNA formyltransferase	0.00E+00	1.44E+03	5.05E+03	-1.00
227	Q1PVQ2	Similar to small heat shock protein	0.00E+00	1.34E+02	5.94E+02	-1.00
228	Q1PXG9	Small heat shock protein-like protein	0.00E+00	2.33E+02	1.20E+03	-1.00
229	Q1Q4D3	TldD/PmbA family protein	0.00E+00	5.86E+01	3.30E+02	-1.00
230	Q1Q4X7	Uncharacterized protein	0.00E+00	3.45E+01	2.33E+02	-1.00
231	Q1Q2M4	Uncharacterized protein	0.00E+00	1.34E+02	1.78E+02	-1.00
232	Q1PY07	Uncharacterized protein	0.00E+00	1.42E+01	1.71E+01	-1.00
233	Q1PW64	Zinc ribbon domain protein	0.00E+00	6.52E+01	3.82E+02	-1.00
