# Supplementary material for: Nitric oxide‐forming nitrite reductases in the anaerobic ammonium oxidizer Kuenenia stuttgartiensis
Source: FEBS Open Bio. 2025 Aug 4;15(10):1696–713. doi: 10.1002/2211-5463.70086 (PMC12485887; doi:10.1002/2211-5463.70086)
Supplement: Supplementary file 7 — Table S5. Correlation between the relative abundance of identified proteins and specific nitrite reductase activity in all fractions obtained with mixed‐mode column chromatography. [file FEB4-15-1696-s003.pdf]

Supplementary table 5 - Correlation between the relative abundance of identified proteins and specific nitrite reductase activity in all fractions obtained with mixed-mode column chromatography. Pearson correlation score was calculated between the specific activity measured per fraction and the relative protein abundance measured per fraction. The data is ordered based on relative protein abundance that correlated best with the specific activity. The relative abundance of HAOr correlated with specific nitrite reductase activity, while NirS does not. Other proteins that showed a positive correlation are not identified as nitrite reductases. Accession numbers refer to the *K. stuttgartiensis* protein sequence database in Uniprot (entry KSMBR1).

Protein number	Accession	Description	Relative protein abundance per fraction				Pearson correlation score
			UV-peak 1	UV-peak 2	UV-peak 3	UV-peak 4	between specific activity and relative protein abundance
1	A0A2C9CHX1	strongly similar to 3naS ribosomal protein S1na	4.75E+04	0.00E+00	0.00E+00	5.15E+05	1.00
2	Q1Q132	strongly similar to 5naS ribosomal protein L11	1.84E+04	0.00E+00	0.00E+00	2.45E+05	1.00
3	Q1PY08	strongly similar to 5naS ribosomal protein L11	2.78E+04	0.00E+00	0.00E+00	3.70E+05	1.00
4	A0A6G7GP74	Putative cellulose synthase, cyclic-di-GMP-binding regulatory subunit	7.21E+05	0.00E+00	0.00E+00	9.99E+06	1.00
5	A0A2C9CFG9	Helicase ATP-binding domain-containing protein	3.70E+04	0.00E+00	0.00E+00	5.91E+05	1.00
6	A0A2C9CJB5	Hydrazine dehydrogenase	3.90E+04	0.00E+00	0.00E+00	3.29E+05	1.00
7	A0A2C9CJU8	Amine oxidase domain-containing protein	6.74E+03	0.00E+00	0.00E+00	1.28E+05	1.00
8	A0A2C9CJI9	site-specific DNA-methyltransferase (adenine-specific)	3.83E+04	0.00E+00	0.00E+00	1.82E+06	1.00
9	Q1Q5F8	strongly similar to bacterioferritin	9.66E+05	0.00E+00	0.00E+00	1.24E+08	1.00
10	Q1Q7J1	similar to hydroxylamine oxidoreductase hao	1.23E+05	2.41E+05	0.00E+00	1.80E+07	1.00
11	Q1Q5E4	hypothetical proteins	0.00E+00	0.00E+00	0.00E+00	1.04E+05	1.00
12	Q1PZT1	similar to ribosomal protein S21	0.00E+00	0.00E+00	0.00E+00	5.97E+04	1.00
13	A0A2C9CIR3	Nucleoside diphosphate kinase	0.00E+00	0.00E+00	0.00E+00	3.36E+05	1.00
14	Q1PZY9	similar to molybdenum cofactor biosynthesis protein	0.00E+00	0.00E+00	0.00E+00	1.15E+05	1.00
15	A0A2C9CGI1	strongly similar to peroxiredoxin (thioredoxin peroxidase)	0.00E+00	0.00E+00	0.00E+00	8.48E+04	1.00
16	Q1Q0K4	hypothetical protein	0.00E+00	0.00E+00	0.00E+00	7.32E+05	1.00
17	Q1Q5P1	similar to naD(P) oxidoreductase, FAD-containing subunit	0.00E+00	0.00E+00	0.00E+00	2.47E+05	1.00
19	Q1PXG4	hypothetical protein	0.00E+00	0.00E+00	0.00E+00	1.75E+05	1.00
20	Q1Q1U2	Methyltransferase type 11 domain-containing protein	1.44E+04	0.00E+00	0.00E+00	7.15E+04	0.99
21	Q1PY40	TIGR04076 family protein	0.00E+00	2.39E+04	0.00E+00	3.21E+05	0.99
22	Q1Q4R9	hypothetical protein	8.45E+04	0.00E+00	0.00E+00	3.88E+05	0.99
**23**	**Q1PVE0**	**similar to hydroxylamine oxidoreductase, HAOr**	**1.51E+04**	**0.00E+00**	**0.00E+00**	**6.86E+04**	**0.99**
24	A0A2C9CFG1	strongly similar to 6na kDa chaperonin (GroEL protein)	4.51E+05	0.00E+00	0.00E+00	1.97E+06	0.99
25	Q1PYJ8	hypothetical protein	3.51E+05	0.00E+00	5.81E+06	6.67E+07	0.99
26	Q1PVE1	c-type diheme-containing protein, redox partner of kustcna458	2.31E+04	0.00E+00	0.00E+00	8.69E+04	0.98
27	Q1Q2J0	similar to molybdenum cofactor biosynthesis protein B	5.04E+03	0.00E+00	2.35E+05	1.85E+06	0.98
28	Q1Q1C8	hypothetical protein	3.08E+04	0.00E+00	0.00E+00	1.09E+05	0.98
29	A0A2C9CDA8	Adenylyl-sulfate kinase	0.00E+00	0.00E+00	6.35E+05	4.36E+06	0.97
30	Q1Q3W5	hypothetical protein	3.46E+05	6.30E+04	0.00E+00	7.92E+05	0.94
31	A0A2C9CF54	hypothetical protein	2.06E+05	0.00E+00	0.00E+00	4.53E+05	0.93
32	Q1PXH4	hypothetical protein	4.93E+06	3.04E+05	0.00E+00	1.00E+07	0.92
33	Q1PUY9	UPF0234 protein KsCSTR_01580	2.17E+04	0.00E+00	0.00E+00	3.09E+04	0.81
34	Q1PYP7	rna binding protein	1.07E+06	0.00E+00	0.00E+00	1.48E+06	0.80
35	A0A2C9CGH9	hypothetical protein	3.90E+06	0.00E+00	0.00E+00	5.22E+06	0.78
36	A0A2C9CJX5	hypothetical protein	6.89E+04	0.00E+00	0.00E+00	8.32E+04	0.74
37	A0A2C9CB72	Putative Alkaline phosphatase	1.82E+06	0.00E+00	0.00E+00	2.14E+06	0.73
38	Q1PZC1	hypothetical protein	3.01E+05	0.00E+00	0.00E+00	3.01E+05	0.64
39	A0A2C9CGA7	4Fe-4S Mo/W bis-MGD-type domain-containing protein	8.39E+04	1.00E+06	0.00E+00	9.90E+05	0.56
40	Q1PXI6	hypothetical protein	0.00E+00	2.08E+06	0.00E+00	1.99E+06	0.54
41	Q1Q0V3	similar to aconitase 1 (aconitate hydratase 1; citrate hydro-lyase 1)	6.50E+05	7.18E+05	2.29E+07	1.92E+07	0.40
42	A0A2C9CJF3	hypothetical protein	1.44E+05	3.02E+06	0.00E+00	2.22E+06	0.38
43	A0A2C9CF13	strongly similar to 6na kDa chaperonin (groEL protein)	2.92E+06	0.00E+00	0.00E+00	1.74E+06	0.35
44	Q1Q2D4	Phosphate-binding protein	1.93E+06	0.00E+00	0.00E+00	1.07E+06	0.31
46	Q1Q131	strongly similar to 5naS ribosomal protein L1	1.53E+06	2.28E+05	0.00E+00	7.76E+05	0.23
47	A0A2C9CDC4	dihydropteroate synthase	2.13E+05	1.41E+05	0.00E+00	1.43E+05	0.22
48	Q1Q782	Hypervirulence associated protein TUDOR domain-containing protein	1.49E+06	5.87E+05	0.00E+00	8.21E+05	0.20
49	Q1Q5X6	hypothetical protein	1.50E+07	2.43E+07	4.50E+08	2.56E+08	0.15
50	Q1PVQ3	similar to small heat shock protein	5.82E+05	2.83E+05	0.00E+00	3.15E+05	0.15
51	Q1PZP6	probable nicotinate-nucleotide adenylyltransferase	3.93E+05	1.11E+06	0.00E+00	5.34E+05	0.06

Protein number	Accession	Description	Relative protein abundance per fraction UV-peak 1	UV-peak 2	UV-peak 3	UV-peak 4	Pearson correlation score between specific activity and relative protein abundance
52	Q1Q0Y1	strongly similar to cytochrome c551 peroxidase	7.60E+06	3.15E+05	0.00E+00	2.28E+06	0.03
53	Q1PXP1	Acetoacetate metabolism regulatory protein AtoC	7.15E+05	0.00E+00	0.00E+00	2.01E+05	0.03
54	A0A2C9CJS6	Vitamin B12-dependent ribonucleotide reductase	2.47E+06	1.23E+05	0.00E+00	6.81E+05	0.01
55	Q1PXV3	Fido domain-containing protein	7.72E+04	1.98E+06	0.00E+00	7.03E+05	0.00
56	Q1PUV5	Methyltransferase domain-containing protein	1.19E+06	0.00E+00	8.92E+05	6.51E+05	-0.01
57	A0A2C9CHM2	hydrazine synthase subunit C	4.25E+05	0.00E+00	0.00E+00	9.13E+04	-0.04
59	A0A2C9CAJ2	Rhamnogalacturonan lyase domain-containing protein	1.69E+07	2.47E+06	0.00E+00	4.33E+06	-0.05
60	A0A2C9CG37	hypothetical protein	4.11E+07	1.16E+06	0.00E+00	7.88E+06	-0.08
61	A0A2C9CKJ6	hypothetical protein	5.63E+05	3.25E+05	0.00E+00	2.17E+05	-0.08
62	Q1Q4B6	hypothetical protein	8.41E+05	0.00E+00	0.00E+00	1.29E+05	-0.10
63	Q1PZD8	nitrite oxidoreductase subunit A	7.25E+06	4.38E+05	0.00E+00	1.13E+06	-0.12
64	A0A2C9CJW9	Macro domain-containing protein	5.68E+06	0.00E+00	0.00E+00	7.36E+05	-0.13
65	A0A2C9CHN2	hydrazine synthase subunit A	1.41E+06	0.00E+00	0.00E+00	1.80E+05	-0.13
66	A0A2C9CB53	Periplasmic zinc binding protein-like protein	8.73E+04	5.19E+06	0.00E+00	1.06E+06	-0.15
67	Q1Q6B5	Cell division protein FtsI (Peptidoglycan synthetase)	1.30E+06	0.00E+00	0.00E+00	1.22E+05	-0.16
68	Q1Q2D9	similar to heat shock protease DegP/HtrA	1.99E+06	0.00E+00	0.00E+00	1.69E+05	-0.17
69	Q1PZD5	nitrite oxidoreductase subunit B	3.19E+06	4.82E+05	0.00E+00	4.63E+05	-0.17
70	A0A2C9CAF9	2-C-methyl-D-erythritol 4-phosphate cytidylyltransferase	8.44E+06	0.00E+00	0.00E+00	6.38E+05	-0.18
71	A0A2C9CDZ7	S-layer protein	9.01E+05	0.00E+00	0.00E+00	6.59E+04	-0.18
72	A0A2C9CCT8	Aldehyde oxidase/xanthine dehydrogenase	1.73E+07	0.00E+00	0.00E+00	1.25E+06	-0.18
73	Q1Q2I9	MOSC domain-containing protein	1.06E+06	0.00E+00	0.00E+00	7.29E+04	-0.19
74	Q1PZD4	nitrite oxidoreductase subunit C	1.61E+06	0.00E+00	0.00E+00	1.09E+05	-0.19
75	A0A2C9CH14	hydrazine synthase subunit B	8.55E+05	0.00E+00	0.00E+00	5.63E+04	-0.19
76	A0A2C9CHG0	hypothetical protein	1.39E+05	4.21E+06	2.42E+05	8.44E+05	-0.19
77	Q1PXR8	strongly similar to aspartate transaminase	2.26E+07	3.73E+05	2.59E+06	2.86E+06	-0.19
**78**	**Q1Q4F5**	**strongly similar to cd1 nitrite reductase NirS**	**1.32E+06**	**1.90E+05**	**0.00E+00**	**1.64E+05**	**-0.19**
79	Q1Q353	DNA polymerase beta	1.62E+06	6.66E+06	0.00E+00	1.55E+06	-0.20
80	Q1Q4U2	Putative methylmalonyl-CoA epimerase	3.04E+06	0.00E+00	0.00E+00	1.78E+05	-0.20
81	A0A2C9CL22	Strongly similar to rhodanese sulfur transferase and phage shock protein pspE	2.24E+06	0.00E+00	0.00E+00	1.29E+05	-0.20
82	A0A2C9CCM4	UDP-N-acetylglucosamine 1-carboxyvinyltransferase	1.16E+05	0.00E+00	0.00E+00	6.49E+03	-0.20
83	Q1PYZ7	hypothetical protein	4.36E+05	0.00E+00	0.00E+00	2.41E+04	-0.20
84	Q1PZY5	strongly similar to cytochrome c peroxidase	9.66E+07	4.10E+06	0.00E+00	6.93E+06	-0.20
85	A0A2C9CJI3	hypothetical protein	1.84E+06	0.00E+00	0.00E+00	8.82E+04	-0.21
86	A0A2C9CKU3	precorrin-2 dehydrogenase	4.00E+06	1.63E+04	0.00E+00	1.43E+05	-0.22
87	Q1Q3G3	similar to matrilysin (metalloproteinase)	8.18E+06	0.00E+00	0.00E+00	2.55E+05	-0.22
88	A0A2C9CEG0	hypothetical protein/ part of the Nxr 'operon'	1.01E+08	6.94E+06	1.57E+06	7.06E+06	-0.22
89	A0A6G7GLF5	S-layer protein	3.44E+06	0.00E+00	0.00E+00	8.39E+04	-0.23
90	Q1Q1X2	Putative mannosyltransferase B	8.84E+05	0.00E+00	0.00E+00	2.15E+04	-0.23
91	Q1PY41	stronlgy similar to 6na kDa chaperonin (GroEL protein)	9.49E+05	9.60E+04	3.41E+06	9.85E+05	-0.23
92	Q1PVK4	similar to phosphoglucomutase	1.58E+07	8.58E+04	0.00E+00	3.76E+05	-0.23
93	Q1Q1B0	hypothetical protein	7.25E+06	0.00E+00	0.00E+00	1.46E+05	-0.23
94	Q1Q2J4	strongly similar to elongation factor Ts (EF-Ts)	3.04E+07	2.62E+05	0.00E+00	6.97E+05	-0.23
95	Q1Q3G2	Phosphoribosylglycinamide formyltransferase	5.25E+07	5.54E+05	0.00E+00	1.24E+06	-0.23
96	Q1PYD2	strongly similar to peptidylprolylisomerase	1.48E+08	1.30E+06	0.00E+00	3.31E+06	-0.23
97	A0A2C9CAG3	HIT domain-containing protein	4.41E+06	1.51E+04	0.00E+00	8.10E+04	-0.24
98	A0A2C9CC36	N-(5'-phosphoribosyl)anthranilate isomerase	3.13E+06	0.00E+00	0.00E+00	5.14E+04	-0.24
99	A0A2C9CDY7	hypothetical protein	1.92E+06	0.00E+00	0.00E+00	2.41E+04	-0.24
100	A0A2C9CIZ7	hypothetical protein	4.47E+08	2.60E+06	0.00E+00	6.67E+06	-0.24
101	Q1Q4J7	strongly similar to UDP-glucuronate 5'-epimerase	0.00E+00	1.44E+06	0.00E+00	1.57E+05	-0.24
102	A0A2C9CET7	similar to high affinity sulfate transporter (plant)	4.75E+06	0.00E+00	0.00E+00	5.32E+04	-0.24
103	Q1Q648	strongly similar to bifunctional methylene-tetrahydrofolate dehydrogenase	1.36E+08	4.49E+06	6.20E+05	3.75E+06	-0.24
104	Q1Q3U7	similar to N-succinyldiaminopimelate aminotransferase	3.42E+08	3.63E+06	7.74E+03	4.88E+06	-0.24
105	Q1PUK7	strongly similar to formyltetrahydrofolate synthetase	2.58E+08	1.69E+07	1.17E+07	1.63E+07	-0.24
106	Q1Q515	DNA-binding protein	1.35E+07	3.33E+05	0.00E+00	2.70E+05	-0.24
107	Q1Q1G1	Orotidine 5'-phosphate decarboxylase	1.28E+08	1.53E+05	0.00E+00	8.79E+05	-0.25

Protein number	Accession	Description	Relative protein abundance per fraction				Pearson correlation score
			UV-peak 1	UV-peak 2	UV-peak 3	UV-peak 4	between specific activity and relative protein abundance
108	A0A2C9CJJ9	NAD-dependent epimerase/dehydratase domain-containing protein	2.77E+07	7.03E+05	8.48E+05	9.22E+05	-0.25
109	A0A2C9CDL3	GTPase	3.77E+07	2.54E+05	0.00E+00	2.96E+05	-0.25
110	Q1Q6P7	HD-GYP domain-containing protein	2.98E+07	8.89E+04	0.00E+00	1.65E+05	-0.25
111	A0A2C9CI57	dITP/XTP pyrophosphatase	8.22E+06	0.00E+00	0.00E+00	2.73E+04	-0.25
112	Q1Q0Y3	Glucose-6-phosphate isomerase	3.30E+06	0.00E+00	0.00E+00	1.09E+04	-0.25
113	Q1Q117	strongly similar to SAICAR synthase	1.37E+08	7.95E+04	0.00E+00	4.20E+05	-0.25
114	A0A2C9CAC1	strongly similar to glutamate-1-semialdehyde aminomutase	5.55E+08	1.73E+07	4.03E+06	1.08E+07	-0.25
115	Q1PUT0	strongly similar to glucose-1-phosphate adenylyltransferase	1.66E+07	0.00E+00	0.00E+00	0.00E+00	-0.25
116	Q1PV39	Secondary thiamine-phosphate synthase enzyme	1.12E+07	0.00E+00	0.00E+00	0.00E+00	-0.25
117	Q1Q0L1	Tetrapyrrole methylase domain-containing protein	9.33E+06	0.00E+00	0.00E+00	0.00E+00	-0.25
118	Q1PZV4	Anthranilate synthase component 1	5.95E+06	0.00E+00	0.00E+00	0.00E+00	-0.25
119	A0A2C9CGY3	Anhydro-N-acetylmuramic acid kinase	4.83E+06	0.00E+00	0.00E+00	0.00E+00	-0.25
120	Q1Q253	UDP-N-acetylenolpyruvoylglucosamine reductase	2.99E+06	0.00E+00	0.00E+00	0.00E+00	-0.25
121	A0A6G7GJQ2	ATPase AAA-type core domain-containing protein	2.77E+06	0.00E+00	0.00E+00	0.00E+00	-0.25
122	A0A6G7GX23	Aspartate aminotransferase family protein	2.19E+06	0.00E+00	0.00E+00	0.00E+00	-0.25
123	A0A2C9CK09	hypothetical protein	2.16E+06	0.00E+00	0.00E+00	0.00E+00	-0.25
124	Q1PZ73	dTDP-4-dehydrorhamnose 3,5-epimerase	2.16E+06	0.00E+00	0.00E+00	0.00E+00	-0.25
125	A0A6G7GU28	LamG-like jellyroll fold domain-containing protein	1.90E+06	0.00E+00	0.00E+00	0.00E+00	-0.25
126	Q1PVD3	Probable 6-oxopurine nucleoside phosphorylase	1.26E+06	0.00E+00	0.00E+00	0.00E+00	-0.25
127	A0A2C9CCA4	hypothetical protein	1.16E+06	0.00E+00	0.00E+00	0.00E+00	-0.25
128	Q1Q0Z5	hypothetical protein	1.08E+06	0.00E+00	0.00E+00	0.00E+00	-0.25
129	Q1PZ46	similar to RND multidrug efflux membrane fusion protein MexC precursor	8.41E+05	0.00E+00	0.00E+00	0.00E+00	-0.25
130	A0A2C9CHD9	strongly similar to dihydrodipicolinate reductase	8.26E+05	0.00E+00	0.00E+00	0.00E+00	-0.25
131	Q1PXP9	Protein-glutamate methylesterase/protein-glutamine glutaminase	7.90E+05	0.00E+00	0.00E+00	0.00E+00	-0.25
132	A0A2C9CCG0	hypothetical protein	6.76E+05	0.00E+00	0.00E+00	0.00E+00	-0.25
133	A0A2C9CGM9	hypothetical protein	5.53E+05	0.00E+00	0.00E+00	0.00E+00	-0.25
134	Q1PYC5	Hydrazine dehydrogenase	4.84E+05	0.00E+00	0.00E+00	0.00E+00	-0.25
135	Q1Q7Q7	hypothetical protein	4.81E+05	0.00E+00	0.00E+00	0.00E+00	-0.25
136	Q1Q0A1	similar to serine-proteinase HtrA/ DegQ/ DegS family protein	4.27E+05	0.00E+00	0.00E+00	0.00E+00	-0.25
137	A0A2C9CJS3	hypothetical protein	3.91E+05	0.00E+00	0.00E+00	0.00E+00	-0.25
138	Q1Q4R8	strongly similar to chorismate mutase / prephenate dehydratase	3.91E+05	0.00E+00	0.00E+00	0.00E+00	-0.25
139	A0A6G7GY31	hypothetical protein	3.89E+05	0.00E+00	0.00E+00	0.00E+00	-0.25
140	Q1PXY8	hypothetical protein	3.04E+05	0.00E+00	0.00E+00	0.00E+00	-0.25
141	A0A2C9CBC8	Formiminotetrahydrofolate cyclodeaminase	1.90E+05	0.00E+00	0.00E+00	0.00E+00	-0.25
142	A0A2C9CBD6	Putative enzyme	1.49E+05	0.00E+00	0.00E+00	0.00E+00	-0.25
143	A0A2C9CGE2	Strongly similar to NAD(P)H:quinone oxidoreductase chain 5	1.04E+05	0.00E+00	0.00E+00	0.00E+00	-0.25
144	A0A2C9CCG2	hypothetical protein	1.03E+05	0.00E+00	0.00E+00	0.00E+00	-0.25
145	A0A2C9CC83	strongly similar to 3naS ribosomal protein S2	9.27E+04	0.00E+00	0.00E+00	0.00E+00	-0.25
146	A0A2C9CKI0	Adenylyl-sulfate kinase	5.15E+04	0.00E+00	0.00E+00	0.00E+00	-0.25
147	Q1Q1N0	Undecaprenyl phosphate-alpha-4-amino-4-deoxy-L-arabinose transferase	3.78E+04	0.00E+00	0.00E+00	0.00E+00	-0.25
148	A0A2C9CFR0	glutamate formimidoyltransferase	1.69E+07	0.00E+00	0.00E+00	0.00E+00	-0.25
149	A0A2C9CIE4	Transposase IS204/IS1001/IS1096/IS1165 DDE domain-containing protein	5.77E+06	0.00E+00	0.00E+00	0.00E+00	-0.25
150	A0A2C9CLN1	Amidohydrolase-related domain-containing protein	5.44E+06	0.00E+00	0.00E+00	0.00E+00	-0.25
151	A0A2C9CEK8	similar to D-3-phosphoglycerate dehydrogenase (PGDH)	3.32E+06	0.00E+00	0.00E+00	0.00E+00	-0.25
152	Q1Q3C3	hypothetical protein	2.99E+06	0.00E+00	0.00E+00	0.00E+00	-0.25
153	Q1PXZ8	Polar-differentiation response regulator DivK	2.95E+06	0.00E+00	0.00E+00	0.00E+00	-0.25
154	A0A2C9CE43	hypothetical protein	2.52E+06	0.00E+00	0.00E+00	0.00E+00	-0.25
155	A0A2C9CH82	similar to flavoproteins norVW and fprA	2.29E+06	0.00E+00	0.00E+00	0.00E+00	-0.25
156	A0A2C9CIJ9	GGDEF domain-containing protein	2.25E+06	0.00E+00	0.00E+00	0.00E+00	-0.25
157	Q1PZ80	strongly similar to phosphoglycerate kinase	2.12E+06	0.00E+00	0.00E+00	0.00E+00	-0.25
158	A0A2C9CDM5	Glutamine--fructose-6-phosphate aminotransferase [isomerizing]	1.93E+06	0.00E+00	0.00E+00	0.00E+00	-0.25
159	A0A2C9CIF4	Transposase IS200 like protein	1.77E+06	0.00E+00	0.00E+00	0.00E+00	-0.25
160	A0A2C9CKJ5	Gluconeogenesis factor	1.66E+06	0.00E+00	0.00E+00	0.00E+00	-0.25
161	Q1PXW3	strongly similar to ATP-dependent protease La	1.58E+06	0.00E+00	0.00E+00	0.00E+00	-0.25
162	A0A2C9CHA0	strongly similar to 5naS ribosomal protein L7/L12	1.29E+06	0.00E+00	0.00E+00	0.00E+00	-0.25

Protein number	Accession	Description	Relative protein abundance per fraction				Pearson correlation score
			UV-peak 1	UV-peak 2	UV-peak 3	UV-peak 4	between specific activity and relative protein abundance
163	A0A2C9CKL5	Regulator of chromosome condensation (RCC1) repeat protein	1.27E+06	0.00E+00	0.00E+00	0.00E+00	-0.25
164	A0A2C9CDL6	hypothetical protein	1.23E+06	0.00E+00	0.00E+00	0.00E+00	-0.25
165	A0A2C9CK66	Multifunctional fusion protein	1.19E+06	0.00E+00	0.00E+00	0.00E+00	-0.25
166	Q1Q4S6	hypothetical protein	9.71E+05	0.00E+00	0.00E+00	0.00E+00	-0.25
167	Q1PZE3	Carboxypeptidase regulatory-like domain-containing protein	9.55E+05	0.00E+00	0.00E+00	0.00E+00	-0.25
168	A0A2C9CCR3	hypothetical protein	9.42E+05	0.00E+00	0.00E+00	0.00E+00	-0.25
169	Q1PUH0	hypothetical protein	9.15E+05	0.00E+00	0.00E+00	0.00E+00	-0.25
170	Q1Q6W7	Putative mannose-1-phosphate guanylyltransferase	8.96E+05	0.00E+00	0.00E+00	0.00E+00	-0.25
171	Q1PX51	DUF3368 domain-containing protein	8.77E+05	0.00E+00	0.00E+00	0.00E+00	-0.25
172	Q1Q3Y4	Purine nucleoside phosphorylase	8.74E+05	0.00E+00	0.00E+00	0.00E+00	-0.25
173	A0A2C9CK74	Cobalamin biosynthesis precorrin-8X methylmutase CobH protein	8.62E+05	0.00E+00	0.00E+00	0.00E+00	-0.25
174	A0A2C9CHW3	Chorismate synthase	8.60E+05	0.00E+00	0.00E+00	0.00E+00	-0.25
175	Q1Q325	3-oxoacyl-[acyl-carrier-protein] reductase	8.25E+05	0.00E+00	0.00E+00	0.00E+00	-0.25
176	Q1Q3U9	hypothetical protein	7.91E+05	0.00E+00	0.00E+00	0.00E+00	-0.25
177	Q1PV08	hypothetical protein	7.55E+05	0.00E+00	0.00E+00	0.00E+00	-0.25
178	Q1PZK1	hypothetical protein	7.45E+05	0.00E+00	0.00E+00	0.00E+00	-0.25
179	A0A2C9CBT8	ABC transporter domain-containing protein	7.41E+05	0.00E+00	0.00E+00	0.00E+00	-0.25
180	A0A2C9CBS9	strongly similar to aspartate-semialdehyde dehydrogenase Asd	7.29E+05	0.00E+00	0.00E+00	0.00E+00	-0.25
181	A0A2C9CJI8	similar to aminopeptidase A	6.79E+05	0.00E+00	0.00E+00	0.00E+00	-0.25
182	A0A2C9CCE6	HD-GYP domain-containing protein	6.08E+05	0.00E+00	0.00E+00	0.00E+00	-0.25
183	Q1Q6N2	Transposase IS4-like domain-containing protein	6.00E+05	0.00E+00	0.00E+00	0.00E+00	-0.25
184	Q1Q1A6	hypothetical protein	5.47E+05	0.00E+00	0.00E+00	0.00E+00	-0.25
185	Q1Q156	strongly similar to adenylate kinase	4.45E+05	0.00E+00	0.00E+00	0.00E+00	-0.25
186	Q1PVS7	Metallo-beta-lactamase family protein, RNA-specific	4.38E+05	0.00E+00	0.00E+00	0.00E+00	-0.25
187	Q1Q3W8	hypothetical protein	3.70E+05	0.00E+00	0.00E+00	0.00E+00	-0.25
188	Q1PZ79	Ribulose-phosphate 3-epimerase	3.63E+05	0.00E+00	0.00E+00	0.00E+00	-0.25
189	Q1PZ47	DNA-(apurinic or apyrimidinic site) lyase	3.00E+05	0.00E+00	0.00E+00	0.00E+00	-0.25
190	Q1PX52	Ribbon-helix-helix protein CopG domain-containing protein	2.96E+05	0.00E+00	0.00E+00	0.00E+00	-0.25
191	Q1PY25	strongly similar to thioredoxin	2.67E+05	0.00E+00	0.00E+00	0.00E+00	-0.25
192	Q1Q247	Small ribosomal subunit protein bS16	2.57E+05	0.00E+00	0.00E+00	0.00E+00	-0.25
193	Q1Q1K7	Ferric uptake regulation protein FUR	2.48E+05	0.00E+00	0.00E+00	0.00E+00	-0.25
194	Q1PUZ1	strongly similar to 6-phosphogluconate dehydrogenase (decarboxylating)	2.36E+05	0.00E+00	0.00E+00	0.00E+00	-0.25
195	Q1Q5Y4	Reverse transcriptase domain-containing protein	2.27E+05	0.00E+00	0.00E+00	0.00E+00	-0.25
196	Q1PY42	strongly similar to 1na kDa chaperonin (GroES protein)	2.09E+05	0.00E+00	0.00E+00	0.00E+00	-0.25
197	Q1PUZ9	hypothetical protein	2.08E+05	0.00E+00	0.00E+00	0.00E+00	-0.25
198	A0A2C9CFP5	Transposase (putative) YhgA-like domain-containing protein	1.69E+05	0.00E+00	0.00E+00	0.00E+00	-0.25
199	A0A2C9CFA5	strongly similar to pyrroline-5-carboxylate reductase	1.56E+05	0.00E+00	0.00E+00	0.00E+00	-0.25
200	Q1Q5M6	hypothetical protein	1.47E+05	0.00E+00	0.00E+00	0.00E+00	-0.25
201	Q1Q4Q2	strongly similar to Dna binding protein HU	1.17E+05	0.00E+00	0.00E+00	0.00E+00	-0.25
202	A0A2C9CH33	Strongly similar to molybdenum cofactor biosynthesis protein C	1.16E+05	0.00E+00	0.00E+00	0.00E+00	-0.25
203	Q1Q205	hypothetical protein	1.05E+05	0.00E+00	0.00E+00	0.00E+00	-0.25
204	A0A2C9CH91	hypothetical protein	1.04E+05	0.00E+00	0.00E+00	0.00E+00	-0.25
205	Q1PX08	strongly similar to nitrogen regulatory protein P-II	9.15E+04	0.00E+00	0.00E+00	0.00E+00	-0.25
206	Q1PXM3	Response regulatory domain-containing protein	8.53E+04	0.00E+00	0.00E+00	0.00E+00	-0.25
207	Q1Q6A7	strongly similar to cold shock protein A	7.96E+04	0.00E+00	0.00E+00	0.00E+00	-0.25
208	Q1PYG6	hypothetical protein	6.33E+04	0.00E+00	0.00E+00	0.00E+00	-0.25
209	Q1PX26	hypothetical protein	5.40E+04	0.00E+00	0.00E+00	0.00E+00	-0.25
210	Q1PYI8	strongly similar to alanyl-tRna synthetase	3.90E+04	0.00E+00	0.00E+00	0.00E+00	-0.25
211	A0A6G7GJL0	DUF3368 domain-containing protein	3.42E+04	0.00E+00	0.00E+00	0.00E+00	-0.25
212	A0A6G7GSL2	Putative divergent AAA domain family	2.86E+04	0.00E+00	0.00E+00	0.00E+00	-0.25
213	A0A2C9CKH9	Hypoxanthine phosphoribosyltransferase	2.09E+04	0.00E+00	0.00E+00	0.00E+00	-0.25
214	A0A2C9CL87	similar to UDP-glucose 4-epimerase	6.30E+06	0.00E+00	0.00E+00	0.00E+00	-0.25
215	A0A2C9CME9	Nitroreductase domain-containing protein	1.13E+06	0.00E+00	0.00E+00	0.00E+00	-0.25
216	Q1Q1J8	Catecholate siderophore receptor CirA	4.34E+04	0.00E+00	0.00E+00	0.00E+00	-0.25
217	Q1PYG4	GDP-L-fucose synthase	1.44E+04	0.00E+00	0.00E+00	0.00E+00	-0.25

Protein number	Accession	Description	Relative protein abundance per fraction UV-peak 1	UV-peak 2	UV-peak 3	UV-peak 4	Pearson correlation score between specific activity and relative protein abundance
218	Q1PZT0	Anthranilate phosphoribosyltransferase	9.59E+06	2.28E+04	0.00E+00	0.00E+00	-0.25
219	A0A2C9CIN1	hypothetical protein	6.63E+06	4.66E+04	0.00E+00	1.40E+04	-0.25
220	Q1Q2R9	Putative sorbitol dehydrogenase	3.42E+08	5.58E+06	4.32E+06	4.34E+06	-0.25
221	Q1PZ82	strongly similar to glyceraldehyde-3-phosphate dehydrogenase	1.68E+08	2.53E+07	0.00E+00	1.11E+07	-0.25
222	Q1PZC7	4-diphosphocytidyl-2-C-methyl-D-erythritol kinase	4.04E+07	2.93E+05	0.00E+00	4.10E+04	-0.25
223	Q1PYA3	Putative beta-lactamase-inhibitor-like PepSY-like domain-containing protein	1.27E+07	3.60E+05	0.00E+00	9.86E+04	-0.26
224	Q1Q013	strongly similar to phosphomannomutase	1.01E+08	1.08E+07	0.00E+00	3.88E+06	-0.26
225	Q1Q1I6	Benzoyl-CoA reductase subunit BadG	7.81E+06	0.00E+00	3.66E+06	1.67E+06	-0.26
226	Q1Q5S6	3-phosphoshikimate 1-carboxyvinyltransferase	4.57E+06	1.28E+05	0.00E+00	0.00E+00	-0.26
227	Q1Q6L2	Glycogen synthase	9.63E+06	4.21E+05	0.00E+00	6.42E+04	-0.26
228	A0A2C9CMF8	hypothetical protein	7.62E+05	2.58E+04	0.00E+00	0.00E+00	-0.27
229	Q1PY54	Methyltransferase domain-containing protein	1.13E+06	4.52E+04	0.00E+00	0.00E+00	-0.27
230	Q1Q468	Exported protein	4.83E+07	2.41E+06	0.00E+00	1.38E+05	-0.27
231	A0A2C9CD72	hypothetical protein	2.12E+06	1.40E+05	0.00E+00	0.00E+00	-0.28
232	A0A2C9CKP3	Cobalt-precorrin-3b C17-methyltransferase	2.31E+06	1.60E+05	0.00E+00	0.00E+00	-0.28
233	Q1PV12	Aminotransferase	8.43E+07	9.90E+06	0.00E+00	8.63E+05	-0.29
234	Q1Q637	hypothetical protein	1.23E+08	1.50E+07	2.23E+06	2.72E+06	-0.29
235	A0A2C9CE24	strongly similar to serine hydroxymethyl transferase SHMT	3.12E+08	6.05E+07	1.26E+07	1.89E+07	-0.30
236	Q1Q724	similar to serine protease Do	1.93E+06	2.23E+05	0.00E+00	0.00E+00	-0.30
237	A0A2C9CGA0	Small heat shock protein-like protein	1.08E+06	2.56E+05	0.00E+00	5.78E+04	-0.30
238	Q1PZJ0	similar to flagellar motor protein MotB	2.24E+06	2.64E+05	0.00E+00	0.00E+00	-0.30
239	Q1Q4B8	Nucleotidyl transferase AbiEii/AbiGii toxin family protein	9.45E+06	8.81E+05	2.53E+05	2.33E+04	-0.30
240	Q1Q7N4	similar to (3R)-hydroxymyristoyl acyl carrier protein dehydrase	0.00E+00	6.34E+05	0.00E+00	1.90E+04	-0.32
241	A0A2C9CFE9	Fragment of pimeloyl-CoA synthetase (Part 1)	4.65E+06	7.42E+05	0.00E+00	0.00E+00	-0.32
242	A0A2C9CCN1	Fragment of pimeloyl-CoA synthetase (Part 2)	4.75E+06	7.59E+05	0.00E+00	0.00E+00	-0.32
243	Q1PXW4	N-acetyl-gamma-glutamyl-phosphate reductase	1.29E+07	3.87E+06	0.00E+00	7.60E+05	-0.32
244	A0A2C9CBM9	Fibronectin type-III domain-containing protein	4.50E+06	7.51E+05	0.00E+00	0.00E+00	-0.32
245	Q1PY88	similar to superoxide dismutase	5.88E+07	1.33E+06	9.88E+06	1.25E+06	-0.33
246	Q1PZC8	similar to unknown protein involved in septum location	2.01E+06	5.07E+05	0.00E+00	6.89E+04	-0.33
247	A0A2C9CFE7	Thymidylate kinase	2.21E+06	6.71E+05	0.00E+00	1.23E+05	-0.33
248	A0A2C9CJJ8	Exoribonuclease YhaM-like protein	1.33E+06	2.35E+05	0.00E+00	0.00E+00	-0.33
249	A0A2C9CJE3	hypothetical protein	1.22E+07	2.60E+06	0.00E+00	1.56E+05	-0.33
250	A0A2C9CHS3	similar to 3-isopropylmalate dehydratase, large subunit	1.88E+06	3.54E+05	0.00E+00	0.00E+00	-0.33
251	Q1Q1N5	Acetate-CoA ligase [ADP-forming] I	1.09E+08	2.19E+07	3.56E+05	4.66E+05	-0.33
252	Q1Q4S4	L,D-transpeptidase YkuD	1.89E+07	4.05E+06	0.00E+00	1.18E+05	-0.34
253	A0A2C9CLA0	Methionyl-tRNA formyltransferase	2.95E+06	9.82E+06	1.67E+05	1.24E+06	-0.34
254	A0A2C9CIL6	Phospholipase D-like domain-containing protein	0.00E+00	5.93E+06	0.00E+00	0.00E+00	-0.34
255	A0A6G7GQY5	hypothetical protein	0.00E+00	3.83E+05	0.00E+00	0.00E+00	-0.34
256	A0A2C9CHK8	DUF11 domain-containing protein	0.00E+00	6.75E+06	0.00E+00	0.00E+00	-0.34
257	Q1PXN9	Putative purine	0.00E+00	1.88E+06	0.00E+00	0.00E+00	-0.34
258	Q1PZ64	Competence protein ComM	0.00E+00	1.45E+06	0.00E+00	0.00E+00	-0.34
259	Q1PV42	similar to naD(P) oxidoreductase, FAD-containing subunit	0.00E+00	8.21E+05	0.00E+00	0.00E+00	-0.34
260	Q1Q5P4	Putative desampylase	0.00E+00	6.45E+05	0.00E+00	0.00E+00	-0.34
261	A0A2C9CG62	DUF262 domain-containing protein	0.00E+00	3.04E+05	0.00E+00	0.00E+00	-0.34
262	A0A2C9CF87	TonB-dependent receptor	0.00E+00	3.03E+05	0.00E+00	0.00E+00	-0.34
263	Q1PXU8	hypothetical protein	0.00E+00	1.49E+05	0.00E+00	0.00E+00	-0.34
264	Q1PVL5	Helicase C-terminal domain-containing protein	0.00E+00	2.85E+06	0.00E+00	0.00E+00	-0.34
265	A0A2C9CFN1	Aminoglycoside phosphotransferase domain-containing protein	0.00E+00	1.05E+06	0.00E+00	0.00E+00	-0.34
266	A0A2C9CCF9	hypothetical protein	0.00E+00	2.13E+05	0.00E+00	0.00E+00	-0.34
267	A0A2C9CI35	hypothetical protein	8.43E+06	4.53E+06	0.00E+00	1.03E+06	-0.35
268	A0A2C9CAV3	hypothetical protein	1.32E+06	2.82E+07	3.77E+05	2.75E+05	-0.36
269	Q1Q5R5	Aspartokinase	1.64E+06	4.63E+05	0.00E+00	2.53E+03	-0.37
270	Q1Q0T9	hypothetical (triheme) protein	1.60E+06	1.29E+07	5.08E+09	1.65E+08	-0.37
271	Q1PUI8	Thiamine-phosphate synthase	1.89E+05	5.32E+05	0.00E+00	4.61E+04	-0.38
272	Q1PY86	hypothetical protein	4.21E+06	3.76E+06	0.00E+00	7.09E+05	-0.39

Protein number	Accession	Description	Relative protein abundance per fraction				Pearson correlation score
			UV-peak 1	UV-peak 2	UV-peak 3	UV-peak 4	between specific activity and relative protein abundance
273	Q1Q277	hypothetical protein	2.31E+06	8.37E+05	0.00E+00	2.67E+04	-0.39
274	Q1Q1K9	hypothetical protein	6.95E+06	1.40E+07	1.85E+08	1.31E+07	-0.39
275	A0A2C9CHA8	similar to intracellular proteinase I	2.35E+07	9.38E+07	1.32E+06	4.19E+06	-0.40
276	A0A2C9CAR7	Murein hydrolase activator NlpD	1.30E+06	4.73E+05	0.00E+00	0.00E+00	-0.40
277	Q1PZB4	hypothetical protein	7.79E+05	2.85E+05	0.00E+00	0.00E+00	-0.40
278	A0A2C9CD11	Acetoacetate metabolism regulatory protein AtoC	1.66E+06	1.45E+06	0.00E+00	2.40E+05	-0.40
279	A0A2C9CHE2	strongly similar to glutamine amidotransferase class I	4.93E+05	1.99E+05	0.00E+00	0.00E+00	-0.41
280	A0A2C9CJG4	Dihydrolipoamide acetyltransferase component of pyruvate dehydrogenase	4.19E+04	4.14E+05	1.19E+07	1.20E+04	-0.42
281	A0A2C9CJ81	strongly similar to S-adenosylmethionine synthetase	8.02E+06	3.70E+08	1.84E+08	6.82E+07	-0.42
282	Q1PW67	similar to peptidyl-prolyl cis-trans isomerase	2.23E+04	7.18E+04	0.00E+00	0.00E+00	-0.45
283	Q1Q1P5	Ketoacid-binding protein	1.17E+06	6.66E+05	0.00E+00	0.00E+00	-0.46
284	Q1PY19	tRNA-splicing ligase RtcB	1.11E+07	9.04E+06	0.00E+00	6.57E+05	-0.46
285	A0A2C9CC13	similar to ribosome recycling factor	5.24E+07	9.21E+07	4.93E+04	3.78E+06	-0.47
286	Q1Q2M7	Putative NADP-dependent glyceraldehyde-3-phosphate dehydrogenase GapN	5.49E+06	8.35E+06	0.00E+00	4.30E+05	-0.47
287	Q1Q1G5	strongly similar to translation initiation factor IF-2	1.16E+06	4.65E+06	3.73E+05	0.00E+00	-0.48
288	A0A2C9CLS3	hydroxymethylpyrimidine kinase	7.45E+06	5.45E+06	0.00E+00	9.55E+04	-0.48
289	Q1PUW1	CBS domain-containing protein	7.80E+05	8.18E+05	0.00E+00	0.00E+00	-0.52
290	A0A6G7GXG7	Monoheme cytochrome	3.29E+05	4.24E+05	0.00E+00	0.00E+00	-0.52
291	Q1Q096	Uroporphyrin-III C-methyltransferase	7.04E+04	8.66E+04	0.00E+00	0.00E+00	-0.52
292	A0A2C9CIK3	Outer membrane protein	1.17E+07	1.77E+08	1.32E+08	3.09E+07	-0.54
293	A0A2C9CGT8	c-type diheme-containing protein, redox partner of kustc0458	2.47E+08	9.09E+08	2.58E+08	5.19E+07	-0.57
294	A0A2C9CAZ0	hypothetical protein	1.68E+07	8.17E+06	3.66E+06	2.63E+05	-0.58
295	A0A2C9CH11	Ketol-acid reductoisomerase (NADP(+))	2.61E+07	3.75E+08	2.97E+08	2.01E+07	-0.64
296	A0A2C9CJP1	similar to hydroxylamine oxidoreductase hao	0.00E+00	3.00E+06	3.14E+06	0.00E+00	-0.65
297	Q1Q2C3	Phospholipase D precursor	3.72E+06	2.18E+06	2.73E+06	2.93E+04	-0.88
298	Q1PZ43	Putative pterin-4-alpha-carbinolamine dehydratase	8.91E+05	1.09E+06	1.07E+06	2.87E+05	-0.99
